# Epigenetic profiles of tissue informative CpGs inform ALS disease status and progression

**DOI:** 10.1186/s13073-025-01542-5

**Published:** 2025-10-16

**Authors:** Christa Caggiano, Marco Morselli, Xiaoyu Qian, Barbara Celona, Michael J. Thompson, Shivangi Wani, Anela Tosevska, Kodi Taraszka, Galen Heuer, Shyuan T. Ngo, Frederick J. Steyn, Peter J. Nestor, Leanne Wallace, Pamela McCombe, Susan Heggie, Kathryn Thorpe, Caitlin McElligott, Gemyka English, Anjali Henders, Robert Henderson, Catherine Lomen-Hoerth, Naomi R. Wray, Allan F. McRae, Matteo Pellegrini, Fleur C. Garton, Noah Zaitlen

**Affiliations:** 1 Department of Neurology, UCLA, Los Angeles, CA USA; 2 Institute of Genomic Health, Icahn School of Medicine at Mt Sinai, New York, NY USA; 3 Department of Molecular, Cell, and Developmental Biology, UCLA, Los Angeles, CA USA; 4 Department of Chemistry, Life Sciences, and Environmental Sustainability, University of Parma, Parma, Italy; 5 Institute for Molecular Bioscience, University of Queensland, Brisbane, Australia; 6 Cardiovascular Research Institute, UCSF, San Francisco, CA USA; 7 Systems and Synthetic Biology, Centre for Genomic Regulation, Barcelona, Spain; 8 Department of Internal Medicine III, Division of Rheumatology, Medical University of Vienna, Vienna, Austria; 9 Department of Medical Oncology, Dana-Farber Cancer Institute, Boston, MA USA; 10 Computational and Systems Biology Interdepartmental Program, UCLA, Los Angeles, California USA; 11 Australian Institute for Bioengineering and Nanotechnology, The University of Queensland, Brisbane, Australia; 12 Department of Neurology, Royal Brisbane and Women’s Hospital, Brisbane, QLD Australia; 13 School of Biomedical Sciences, Faculty of Medicine, The University of Queensland, Brisbane, Australia; 14 Queensland Brain Institute, University of Queensland, Brisbane, Australia; 15 Mater Public Hospital, Brisbane, Australia; 16 Department of Neurology, UCSF, San Francisco, California USA; 17 Department of Human Genetics, UCLA, Los Angeles, California USA

**Keywords:** Cell-free DNA, Neurodegeneration, Epigenetics

## Abstract

**Background:**

Cell-free DNA (cfDNA), derived from dying cells, has demonstrated utility across multiple clinical applications. However, its potential in neurodegenerative diseases remains underexplored, with most existing cfDNA technologies tailored to specific disease contexts like cancer or non-invasive prenatal screening.

**Methods:**

To address this gap, we developed a novel approach to characterize epigenetic cfDNA profiles by identifying key regions of DNA methylation that reveal the tissues origins undergoing apoptosis or necrosis. We evaluated this method in the largest cfDNA study of amyotrophic lateral sclerosis (ALS) and other neurological diseases (OND) to date, encompassing two independent cohorts (*n* = 192) from Australia (UQ N_cases_ = 48, N_controls_ = 32, N_OND_ = 15) and the USA, (UCSF N_cases_ = 50, N_controls_ = 45)).

**Results:**

Our approach accurately distinguished ALS patients from controls (UQ AUC = 0.82, UCSF AUC = 0.99) and from individuals with other neurological diseases (AUC = 0.91). It also identified an asymptomatic carrier of a pathogenic *C9orf72* variant, and strongly correlated with ALS disease progression measures (Pearson’s *R* = 0.66, *p* = 3.71 × 10⁻⁹).

**Conclusions:**

We identified DNA methylation signals from multiple tissue types in ALS cfDNA, highlighting diverse tissue involvement in ALS pathology. These findings promote epigenetic cfDNA analysis as a powerful tool for advancing our understanding of neurodegenerative disease.

**Supplementary Information:**

The online version contains supplementary material available at 10.1186/s13073-025-01542-5.

## Background

Cell-free DNA (cfDNA) is a promising biomarker candidate for diverse health outcomes, as it originates from dying tissues and can be non-invasively measured through a blood draw [1]. It has been used to detect cancer [2–4], identify fetal genetic abnormalities [5, 6], screen for infectious diseases [7, 8], and predict pregnancy complications [9]. One underexplored domain for cfDNA, however, is in neurodegenerative disease. While the application of cfDNA to neurodegeneration is nascent, our previous work [10], along with the work of others [11–13], has shown alterations in the cell-free DNA and RNA of patients with neurodegeneration relative to healthy controls.

Here, we build upon this work with a novel approach to characterize the epigenetic cfDNA profile of patients with neurodegenerative disease via DNA methylation. A limitation of whole genome methylation approaches is that the cost to achieve high sequencing coverage [14] is not economical to be routinely applied in clinical settings [3, 15]. However, high sequencing coverage is needed since certain cfDNA fragments may only be present in low quantities. Furthermore, many methylation sites are not variable between tissues, which provides little information about disease state. To address these limitations, previous work has used DNA methylation capture to enrich for relevant genomic regions, which can reduce sequencing costs while maintaining high coverage [4, 16–18]. While these approaches have been successful in specific disease contexts, they have not been generalized for neurodegenerative disease.

In this work, we developed an approach to identify DNA methylation regions that are informative for the presence of specific tissues in cfDNA. These regions can be used to learn about tissue death in a range of diseases, including neurodegeneration. We then developed algorithms that leveraged differences in the methylation state of these tissue informative sites to classify patients by disease status based on their epigenetic cfDNA profile. Our methodology can be used to characterize the contribution of diverse tissues to cfDNA, leading to a multidimensional picture of disease.

We evaluated this technology in a large cohort of amyotrophic lateral sclerosis (ALS) patients, healthy controls, and patients with other neurodegenerative diseases from the University of California at San Francisco, United States (UCSF) and the University of Queensland in Brisbane, Australia (UQ). Together, these cohorts represent the largest application of cfDNA in the study of ALS to date (n = 192). Methylation derived features measured in the cfDNA discriminated ALS patients from controls with high accuracy (UQ AUC = 0.82, UCSF AUC = 0.99), and discriminated ALS patients from those with other neurological diseases (AUC = 0.91). It also identified a previously unknown asymptomatic carrier of a pathogenic variant in *C9orf72,* which is the main monogenic cause of ALS. Additionally, the cfDNA epigenetic features could significantly predict measures of ALS disease progression, (p = 3.71⨉10^–9^). Lastly, we identified important epigenetic regions informative for the presence of a range of tissues in the cfDNA of ALS patients, including skeletal muscle, small intestine, and T-cells, suggesting that multiple sources of tissue degeneration are important to ALS biology. Together, these results highlight cfDNA methylation as a promising quantitative biomarker candidate for ALS.

## Methods

### Patient recruitment and clinical data

A total of 192 participants were enrolled in a prospective manner at the UCSF ALS Clinic in San Francisco, California, USA, the Royal Brisbane and Women’s Hospital and Mater Hospital in Brisbane, Australia under neurologist supervision from 2018–2021. All participants provided written informed consent and the study received approval from the Human Research Ethics Committee at the Royal Brisbane and Women’s Hospital (HREC/17/QRBW/299) and by the UCSF Committee on Human Research (IRB 10–05027). For validation of the capture panel, an additional n = 5 healthy donors were obtained from UCSF.

Patients (with ALS/being assessed for ALS) and when possible, control (non-related, closely age-matched family members, caregivers, or volunteers) were recruited. A second set of other neurological controls were recruited from a non-ALS outpatient clinic under neurologist supervision. Allocation to diagnostic groups was performed according to the latest available clinical information (clinical censor date October 2023).

For cases and controls, age, sex, and self-identified race/ethnicity (SIRE) were recorded. For ALS cases at the time of visit, FVC and ALSFRS-R were taken, and ALSFRS-R slope and FVC slope relative to the previous visit were calculated. The symptom onset site and date of first symptoms were also recorded.

To stabilize the cell-free DNA, all blood samples were collected in the PAXgene Blood ccfDNA Tubes following a clinic appointment. To ensure enough cfDNA was available for downstream applications 20 mL of whole blood from controls/OND and 10 mL of whole blood from cases were collected. Following laboratory receipt (typically within 24-48 h of collection) blood was spun with the brake off (10 min, 1900 g) before plasma was aliquoted and spun twice (10 min, 16000 g) to remove any further debris. Plasma was then stored at −80 before further processing.

### Probe design

Probes were designed to capture TIMs. These were originally defined in our previous work [10]. Individual CpGs were selected if they were informative for tissue deconvolution based on cell-type-specific methylation patterns. TIMs within 500 bp of each other were removed to enrich as many regions as possible in capture.

This resulted in a List of 4,994 TIMs. Using proprietary IDT capture design methods, a methylated and unmethylated probe were designed to bind and capture both possible states of the target CpG. To increase the efficiency of the capture, 120 base pair probes were designed to bind to a window around the TIM. The 120 bp probes were designed by IDT to maximize the experimental capture efficiency of the TIM. During bisulfite conversion, any cytosine base not protected by a methyl group in position 5 is converted into thymine [19]. Since methylation in humans primarily occurs at CpG sites, this means that all cytosines on the forward strand would be converted to thymine. Thus, to capture the unmethylated CpG state, the unmethylated probe was designed with all guanine bases converted to adenine. For the methylated state, where only cytosines in a CpG dinucleotide would be protected from the bisulfite treatment, only non-CpG guanine bases were converted to an adenine.

To examine whether the probes could accurately capture the methylation state of the cfDNA, we profiled pools of universal methylated DNA standards. Mixtures were created where CpGs were, on average, methylated 0, 25, 50, and 100% of the time. The DNA was sheared via sonication to approximate the shorter segments observed in cfDNA. We sequenced these libraries as above. Then, methylation was estimated using BsBolt (for more details see “Bioinformatic Processing”) and compared to the expected proportions.

Further validation was conducted using samples from n = 5 participants. 2 samples were obtained from whole blood, where sheared genomic DNA from blood was used to mimic cfDNA patterns. 3 samples were healthy cfDNA samples from plasma. One additional healthy participant was included for validation. Two plasma samples were extracted from this participant before and after vigorous exercise (running up and down stairs for 20 minutes) and cfDNA was extracted from each timepoint. Each of these 6 samples were sequenced and analyzed identically to the main cohorts.

### Library preparation and sequencing

Using a harmonized protocol across the two sites (UCSF and UQ) cfDNA was extracted and prepared for sequencing. Briefly, plasma was thawed at room temperature and cfDNA was extracted from all available plasma (range 2–8 ml) using the QIAGEN Circulating Nucleic Acid kit (Cat No: 55114) according to the manufacturer's recommendations. Extracted cfDNA was quantified using Qubit dsDNA HS Assay and visualized using the cfDNA assay (Agilent—TapeStation 4200 (UCSF) and Agilent Bioanalyzer 2100 (HS kit) (UQ)). cfDNA was bisulfite converted using the Zymo Lightning kit (Zymo Research) and underwent library preparation using the Accel-NGS Methyl-Seq (Swift Biosciences) according to the manufacturer's instructions with a major modification. Briefly, the denatured BS-converted cfDNA was subject to the adaptase, extension, and ligation reaction. Following the ligation purification, the DNA underwent primer extension (98C for 1 min; 70 C for 2 min; 65 C for 5 min; 4 C hold) using oligos containing random UMI and i5 barcodes. The extension using a UMI-containing primer allows the tagging of each individual molecule in order to be able to remove PCR duplicates and correctly estimate DNA methylation levels.

Following exonuclease I treatment and subsequent purification, the libraries were then amplified using a universal custom P5 primer and custom i7-barcoded P7 primers (initial denaturation: 98 C for 30 s; 15 cycles of: 98 C for 10 s, 60 C for 30 s, 68 C for 60 s; final extension: 68 C for 5 min; 4 C hold). The resulting unique-dual indexed libraries were then purified, quantified using the Qubit HS-dsDNA assay, the quality checked using the D1000-HS assay (Agilent—TapeStation 4200), and grouped as 12-plex pools. Each pool was then subject to hybridization capture using the xGen Hybridization Capture Kit (IDT) using custom probes designed on approximately 5000 pre-selected regions.

Following the hybridization capture, a final amplification PCR (initial denaturation: 98 C for 30 s; 10 cycles of: 98 C for 10 s, 60 C for 30 s, 68 C for 60 s; final extension: 68 C for 5 min; 4 C hold) has been performed, followed by SPRI beads purification and quantification as QC as previously described. To maximize consistency across sites, the same probes were used (shipped to Australia following UCSF library preparation).

The final pool of libraries was submitted for sequencing on an Illumina NovaSeq6000 (USA; UCLA Sequencing facility, Australia;UNSW Ramaciotti Sequencing facility) using identical run conditions (S4 lane—150 PE, 8bases for i7, 17 bases for i5).

### Tissue informative marker selection

WGBS reference data was obtained from BLUEPRINT for hematopoietic cell types, and ENCODE or the International Human Epigenome Consortium (IHEC) (generated specifically from the Canadian Epigenetics, Environment, and Health Research Consortium) [20] for non-hematopoietic cell types, such as organ samples. See Additional file 1: Table 5 for a complete list WGBS samples, their identifiers, and their sources.

TIMs were selected for 18 tissues and cell types: dendritic cells, endothelial cells, eosinophils, erythroblasts, macrophages, monocytes, neutrophils, T-cells, adipose, brain, fibroblast, heart, hepatocytes, lung, megakaryocytes, skeletal muscle, small intestine, and mammary epithelial cells. These tissues were determined based on our previous work to be relevant to ALS (skeletal muscle), or selected based on previous publications on genome-wide cfDNA deconvolution (see Lehmann-Werman et al., [12] Loyfer et al. [21], Moss et al. [22], and Li et al. [23]) to be the plausible contributors to cfDNA. At least two WGBS samples per reference dataset were obtained. The average methylation per CpG for the reference tissue replicates was calculated.

Per CpG, for one tissue at a time, the distance between the methylation proportion at that tissue and the mean methylation of all other tissues was calculated. The *N* sites per tissue with the greatest difference were kept as TIMs. If two tissues had the same CpG classified as a TIM, it was removed from both lists. We focused on single CpGs for initial TIM selection based on our previous TIM selection protocol [10].

To begin, we selected 300 potential TIM sites and then performed quality control checks. To ensure that TIMs were sites that would be covered in cfDNA data, we used two WGBS cfDNA datasets and removed any CpG site that had less than an average of 10X coverage in both datasets. We also removed TIMs that overlapped a common SNP (minor allele frequency > 5%). Since we wanted to have the greatest diversity of regions targeted in the capture, if there were multiple TIM sites within 500 bp of each other, we kept only the first site. This prevented the design of probes that targeted the same region, since many valid TIMs were within 500 bp of each other. Additional quality control was performed to remove TIMs overlapping repetitive regions and with Low predicted target efficiency. After quality control, 4,994 TIMs remained. The code for TIM processing is available at https://github.com/christacaggiano/celfie [24].

### Bioinformatic processing

UMIs for both cohorts were first extracted from the index read and added to the header of the corresponding R1 and R2 fastq file using umi_tools [25]. Aadapters were trimmed using trim_galore. Read alignment, processing, and methylation calling were performed using BsBolt v 1.6.1 [26] in an adapted pipeline published in Morselli et at. [17] Reads were aligned to an hg38 bisulfite converted genome, which was generated using the BsBolt Index over an hg38 fasta file obtained from the UCSC genome browser. Reads were aligned using BsBolt Align in paired end mode with default parameters. To prepare for duplicate removal, aligned reads were subject to samtools fixmate and sorted [27]. Umi_tools [25] dedup in paired end mode was used to remove duplicate reads.

For both cohorts, CpG methylation was called using the command BsBolt CallMethylation -BG -CG -remove-ccgg. The CG parameter restricted to only CpG sites (ignoring non-CpG methylation), the the BG parameter sent the output to a bedgraph file and the -remove-ccgg parameter removed methylation calls in ccgg regions.

### Genetic sex

As a quality control metric, we estimated the genetic sex of the samples and assessed how they corresponded to self-reported sex. We did this using scripts from Phung et al., [28] which calculates the number of reads mapped to chromosome 19 and compares them to the number of reads mapped to the X chromosome. In individuals assigned female at birth, the ratio of chromosome 19 reads should be approximately 1 since they have two X chromosomes and two chromosome 19. We removed one individual whose genetic sex did not match their reported clinical data.

### Deconvolution

cfDNA deconvolution was performed using CelFiE, which is a supervised deconvolution algorithm that is designed for noisy read count data and missing reference tissues. Input sites for CelFiE were the on-target TIMs selected for capture, As demonstrated in the CelFiE publication and in Sun et al., 2015 [29]. from adjacent CpGs can improve deconvolution performance by decreasing sampling noise since CpGs in a small window are locally correlated [30]. As such, reads were summed ± 250 bp around the target CpG. Sites with no reads covering the CpG were set to have a read depth of zero.

Deconvolution was performed using tissues representing organs and hematopoietic cell types, selected for their relevance in cfDNA [10, 22, 31]. CelFiE can estimate an arbitrary number of unknown tissues. Since CelFiE learns from both the input and reference data, the number of samples influences the accuracy of unknown estimation. Based on simulation experiments published in the original CelFiE paper, 2 unknowns were chosen for the sample size of 96 total cfDNA input samples.

The reference panel for CelFiE consisted of 19 tissues over the same on-target TIMs as the input matrix. Reference samples were WGBS samples obtained from ENCODE [32] and Blueprint [33, 34]. Reference samples were also summed in 500 bp regions around the target CpG.

CelFiE was run over the UMI-deduplicated UQ samples, the UMI-deduplicated samples, and both cohorts combined. The CelFIE default of 10 random restarts was used.

After running deconvolution, differences in cell-type proportion between cases and controls were tested for one tissue at a time using the Python StatsModels package. A logistic regression model was run where the outcome was the binary case/control status and the input variable was the estimated tissue of origin proportion for a given tissue. Age, sex, and genetic ancestry were used as covariates.

### Machine learning preprocessing

Samples with more than 10% of targeted CpGs missing, meaning that no reads were covering a CpG, were removed. Any site that had a median read coverage of 1 read or less was also removed. For the remaining sites and samples, the input matrix was made by dividing the number of methylated reads by the total number of reads. Imputation was performed per cohort over the methylation proportion matrix using SoftImpute, implemented in the Python package fancyImpute. For methylation coverage features, the coverage was normalized per sample by dividing the number of reads at a CpG by the total number of sequencing reads per individual. Lastly, sites with Low variance, computed as the bottom 5% of sites in variance, were removed. After filtering 3170 regions remained for input into penalized regression models.

Sex and SIRE were one-hot encoded and added as columns in the input matrix. Age, cfDNA starting concentration, and total cfDNA input were included as continuous covariates. Two separate matrices were kept, one for the ALS case/control status, and one for the methylation proportion and covariates.

### Disease classification

Elastic net regression was performed in R using the BigStatsR package and big_spLogReg command [35]. ALS disease status served as the binary outcome variable, while the DNA methylation proportion at targeted CpGs and clinical variables served as predictors. We incorporated age, genetic sex, SIRE, cfDNA concentration (nanograms/microliter), and total input cfDNA quantity (nanograms) into the regression models as non-penalized variables.

Models were first trained on each cohort separately and then applied to the second cohort. The alpha parameter, which controls model sparsity, was selected by performing ten-fold cross-validation on the training cohort and picking the optimal value. Alpha values evenly spaced between 0 (pure L2 regularization/ridge regression) and 1 (pure L1 regularization/lasso) were supplied to the model for selection. The BigStatsR package removes the manual selection of an optimal lambda value by introducing the Cross-Model Selection and Averaging (CMSA) procedure [35, 36]. In brief, CMSA separates the training set into K folds and then performs cross-validation within the training set to obtain a set of vectors of predictions. This set of coefficients is averaged to produce the final coefficient value. For our model, we used the BigStatR default K value of 10. See Additional file 1: Table 8 for more details on model parameters. To standardize the weights produced per CpG site in each model, we scaled input value parameters to have mean zero and variance one. We scaled the test and training data separately to conservatively prevent data leakage.

Cohort-only models were trained only within a single cohort using ten-fold cross-validation. To evaluate the overall performance of the two cohorts, we trained a single model combining both sets of data and adding the cohort site as a non-penalized covariate. We used generalized linear models with a logit link function and additionally report area under the receiver operator curve (AUC). Code for machine learning applications is available at https://github.com/christacaggiano/cfdna-tims [37].

### Analysis of important features

To examine the importance of important DNA methylation and methylation coverage features in making model predictions, we obtained the weights, or β-values, at each feature from the combined cohort model. We merged the feature β-values with information on what tissue a TIM was selected for and whether it was hyper- or hypo-methylated. We used HOMER [38] to annotate a TIM with the closest gene to the TIM site.

To assess the relationship between the methylation or coverage at a specific TIM site, we performed a logistic regression, with the methylation value of the samples as the predictor and case–control status as the outcome. We used SIRE, age, sex, cfDNA concentration, and total cfDNA input as covariates.

### ALS disease phenotype prediction

ALS disease prediction models were trained for ALSFRS, ALSFRS Slope, and FVC. The top 1000 methylation features and top 1000 coverage features from the combined case–control prediction model were used as input to the model along with age, sex, SIRE, input cfDNA concentration, and total cfDNA input as non-penalized covariates. Due to low sample sizes for the case-only analysis, we meta-analyzed the two cohorts and additionally added cohort as a non-penalized covariate. We trained the elastic net model using the BigStatsR package with the big_spLinReg command. As with the binary disease prediction model, optimal alpha values were selected from a grid of values between 0 and 1. Each of the three models were evaluated against an elastic net model trained on only the covariates.

### ALS disease phenotype prediction

To predict DNA methylation age, the same input TIM sites as were used. A penalized regression model using the BIgStatsR was trained to predict true age, and sex, SIRE, input cfDNA concentration, and total cfDNA input as non-penalized covariates. The model was trained across cohorts to maximize sample size, and a non-penalized covariate of the cohort was included in training. Models were trained using tenfold cross validation. The relationship between estimated DNA methylation age and true age was assessed across participants and cohorts. To assess differences between DNA methylation age and true age, we calculated the “age acceleration” defined as the residual between the predicted and true age. This was to ensure the age acceleration was not dependent on the value of the true age. Age acceleration was calculated using the Python package Statsmodels. To assess the association between age acceleration and ALS disease characteristics, a logistic/linear regression was fit correcting for true age sex, SIRE, input cfDNA concentration, and total cfDNA input.

### Off target prediction models

Off target prediction models incorporated information for all CpGs obtained from high throughput sequencing. Off-target CpGs are those observed not within ± 250 bp of a given TIM, which means they will not be considered for the input into the on-target machine learning models. To do this, we found the union of all sites across all samples in a cohort. To maximize the number of off-target sites considered, we then removed sites with more than 5% missingness. This less rigorous filtering strategy than on-target sites was performed to maximize the number of CpGs retained, since many off-target CpGs were low coverage. Due to the lower coverage and increased number of sites, we did not impute missing sites. Since cohorts had differences in sequencing depth and on-target coverage, sites were analyzed separately. Case control status was then predicted using tenfold cross validation in an elastic net model using the BigStatsR package in the same manner as the on-target models.

### Downsampling simulations

To simulate samples with lower read depth, we used picard DownsampleSam [39] to randomly remove reads at specified proportions of the total starting amount of reads to produce a bam file. We did this for each UCSF cfDNA sample. Then, methylation was re-called on the downsampled bam file using BsBolt to produce a new estimate of the methylation proportion and coverage of a CpG. We then subset to the same CpGs used in the on-target analysis and the same individuals that were used in the on-target analysis, to present an identical setup to that used in Table 2, imputing any missing values with SoftImpute. Then, an elastic net model was trained. The ten fold cross validated AUC was recorded for each set of downsampled samples.

## Results

### Overview of approach

**Fig. 1:**
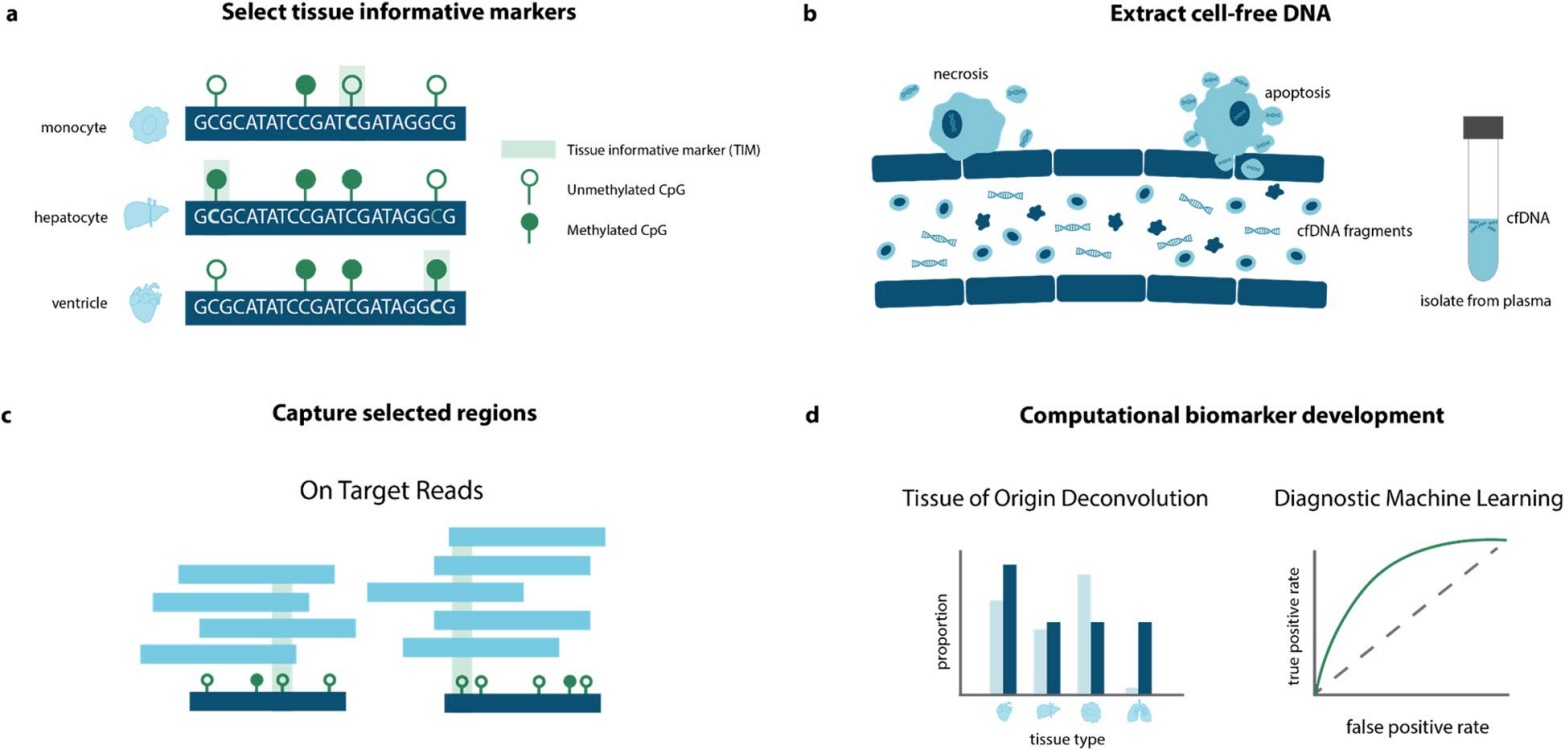
Overview of epigenetic cfDNA biomarker development approach. **a** Firstly, tissue informative markers (TIMs) were selected using WGBS data to capture CpG sites that were hypermethylated or hypomethylated in a tissue of interest using publicly available WGBS reference data. **b** Next, cfDNA was extracted from the blood plasma of ALS cases and controls. **c** The cfDNA was bisulfite-treated, hybridized to capture probes, designed as complementary to TIMs, and then sequenced. Some off-target reads were also captured. **d** Using computational approaches, we analyzed the tissue of origin of the cfDNA samples and performed machine learning to identify features of ALS

The approach was composed of four steps. First, we analyzed publicly available whole-genome bisulfite sequencing (WGBS) tissue data from the ENCODE [32], BLUEPRINT [34], and the International Human Epigenome Consortium (IHEC) [20] databases to identify methylation sites with distinct patterns in a tissue of interest. We call these sites tissue-informative markers (TIMs). Previously published cfDNA WGBS data from diverse contexts, including pregnancy [40] and neurodegenerative disease [10], was used to screen candidate TIMs for those actually observed in cfDNA (See Methods). Probes were designed to capture the TIM regions (Fig. 1a). Next, cfDNA from our two cohorts was extracted, bisulfite converted, (Fig. 1b), and hybridized to the probes. The probe enriched regions were then high-throughput sequenced and their methylation profile was estimated (Fig. 1c) (Methods). Lastly, we analyzed the methylation status of the targeted regions and developed statistical and machine learning approaches to learn about the disease status of the ALS patients and controls (Fig. 1d).

### Cohort characteristics

Our approach was applied to participants (*n* = 192) who were recruited between 2018 and 2021 from two independent university-affiliated neurology clinics at UCSF and UQ (Additional file 1: Table 1). The Revised El Escorial diagnostic criteria [41] were used to classify cases (See Methods). Cases were composed of two groups of patients, those who had likely or probable ALS according to the criteria (referred to here as “ALS”), and those classified as possible ALS or primary lateral sclerosis (PLS) (referred to here as “PLS”), which is a related motor neuron disease [42, 43]. All patients were recruited post-symptomatically.

The UCSF cohort comprised 41 ALS cases, 9 PLS cases, and 45 healthy age-matched controls consisting of unrelated partners or carers. Only 2 patients reported family history of disease (Additional file 1: Table 2). ALS mutation status was unknown for UCSF cases. At UQ, a total of 48 cases were enrolled (*N* = 43 ALS and *N* = 5 PLS). 3 of the UQ cases had confirmed pathogenic mutations in ALS genes, including *c9orf72 and  SOD1* (Additional file 1: Table 3). 6 UQ cases had family history of the disease (Table 2), the remainder were sporadic, or family history was unknown. Forty-eight UQ controls were enrolled, consisting of both unrelated partners/carers (*N* = 32) and patients with other neurological diseases (OND) (*N* = 15). The UQ OND samples included a cross-section of neurological conditions, including diseases that share pathophysiology with ALS, like frontotemporal degeneration [44], and other neurodegenerative diseases like Alzheimer’s disease (Additional file 1: Table 4). Therefore, the UQ cohort represented a challenging real-world scenario for ALS biomarker development.

**Fig. 2:**
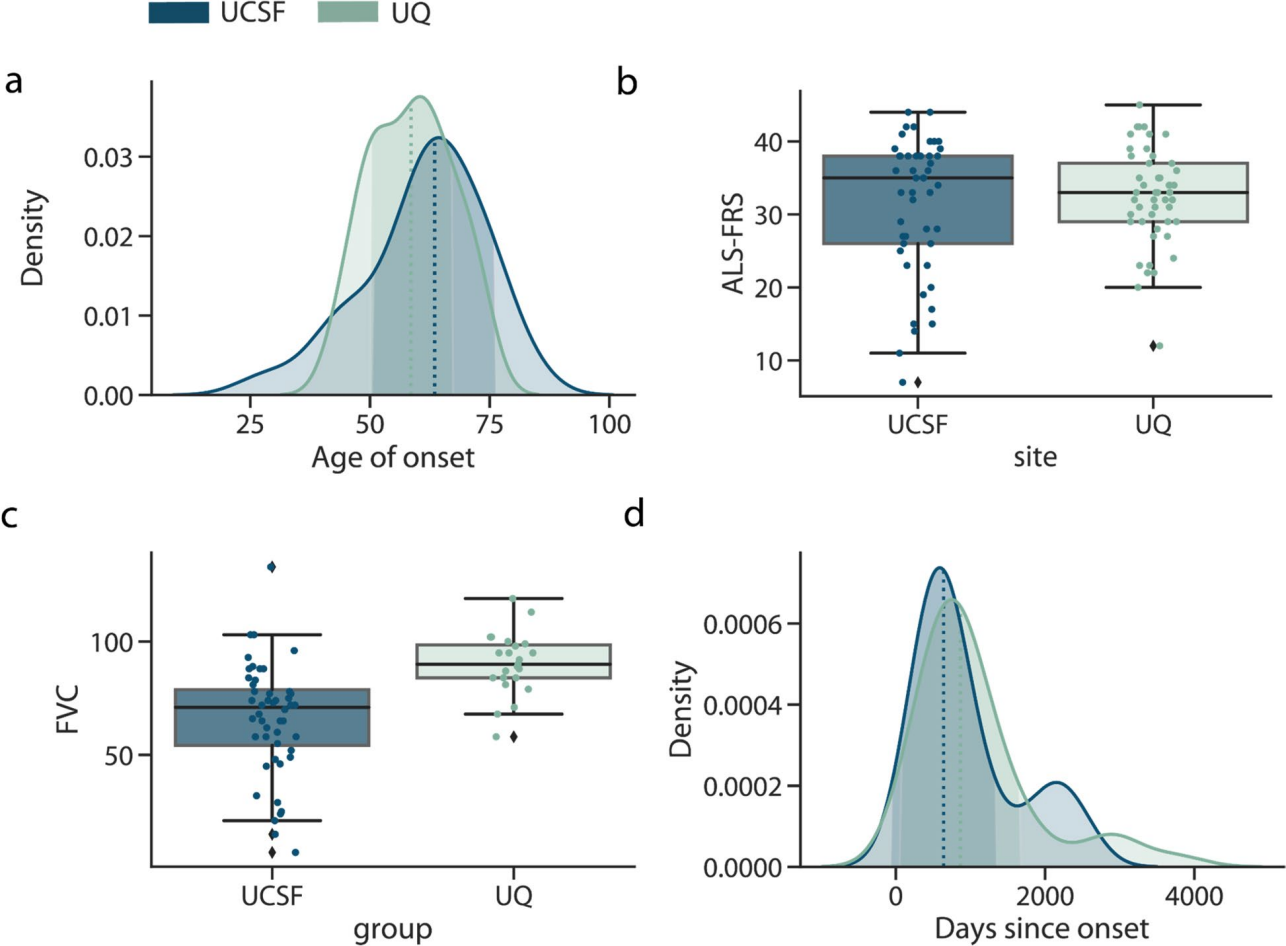
Cohort demographic and clinical characteristics. For the UQ (*n* = 43) and UCSF (*n* = 42) ALS patients. **a** The distribution of the age of onset of ALS disease symptoms, where the dotted line indicates the median age of onset, **b** patient ALSFRS-R scores, **c** FVC, and **d** the number of days between cfDNA collection and date ALS symptoms were observed. In the density plots, the shaded area indicates the continuous probability curve using kernel density estimation. In the box plots, the centerline of the box indicates the mean, the outer edges of the box indicate the upper and lower quartiles, and the whiskers indicate the maxima and minima of the distribution. Each dot indicates an individual

There was heterogeneity of disease characteristics within and between cohorts. Both the UCSF and UQ cases had overlapping distributions in terms of age of onset, defined as the date the first ALS symptom was observed (Fig. 2a). For each cohort, ALS severity was measured using the ALS Functional Rating Scale-Revised (ALSFRS-R) [45] at the time of cfDNA collection, which is a qualitative measure of physical functioning on a scale from 0 (not functioning) to 48 (high functioning). The change in ALSFRS-R between visits, referred to as ALSFRS-R slope, was also calculated as a metric of disease progression. We found that cohorts were similar in the distribution of ALSFRS-R and ALSFRS-R slope, although the UCSF had slightly more progressed cases (Fig. 2b). UQ samples had higher forced vital capacity (FVC) (t-test *p* = 4.5⨉10^–5^), which is a measure of lung function, where a higher value indicates better function (Fig. 2c). The two cohorts were also similar in the distribution of days between cfDNA collection and symptom onset (Fig. 2d). We noted that patients in the UCSF cohort were slightly older (UQ mean age: 61.45 ± 8.17, UCSF mean age: 66.33 ± 9.96)) and that the UCSF cohort also contained patients from a larger variety of self-reported racial and ethnic (SIRE) backgrounds (Additional file 1: Fig. S1b-d).

### Selecting tissue informative markers

**Fig. 3:**
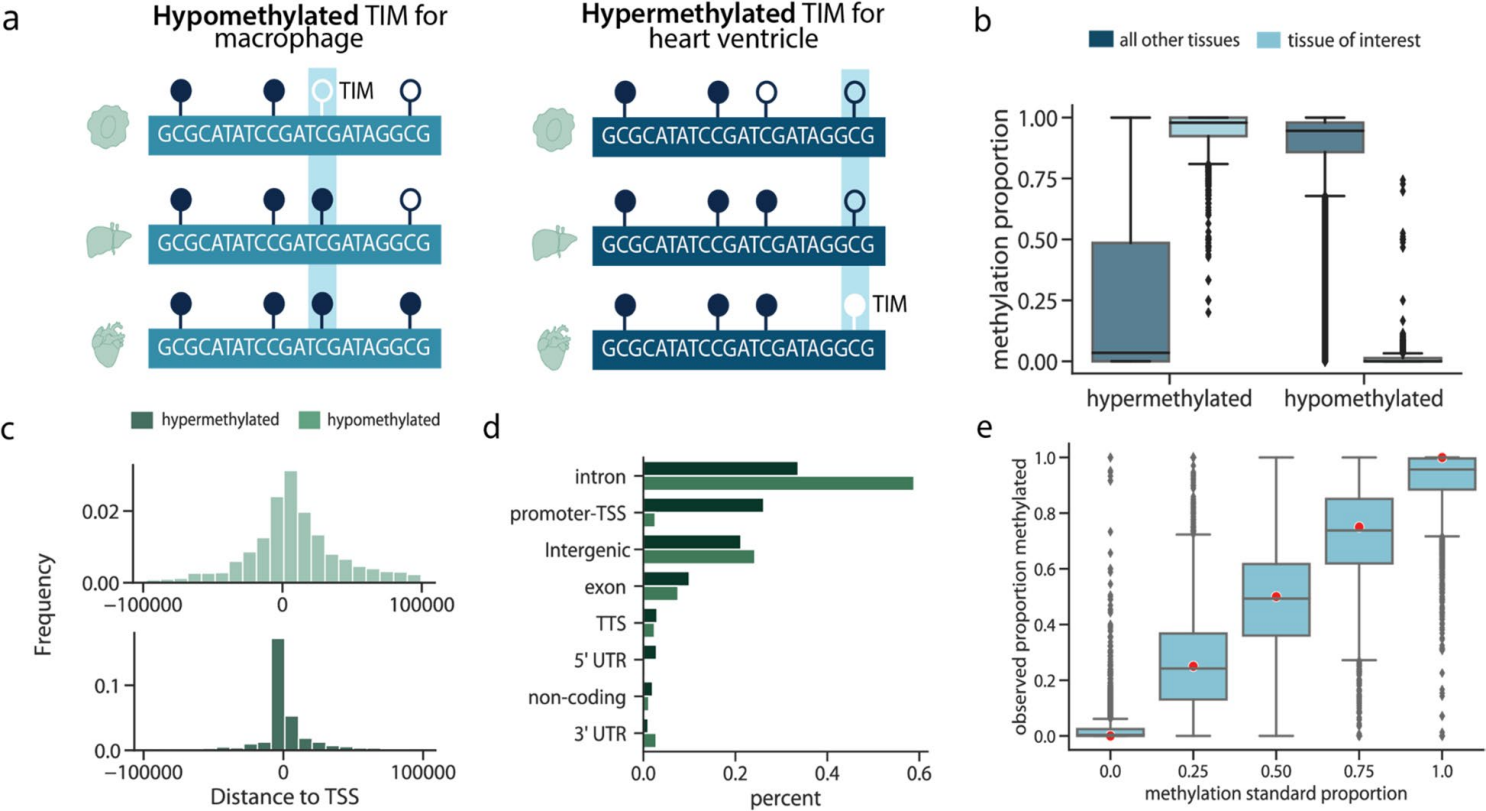
Capture panel design. **a** The panel was designed to capture both hypomethylated TIMs, which were CpG sites that were less methylated in a tissue of interest relative to other tissues, and hypermethylated TIMs, which were designed to capture sites more methylated in a tissue of interest than other tissues. **b** The methylation proportion of reference tissues at either the site the TIM was selected for or all other tissues. **c** The distance hyper- or hypo-methylated TIMs are from the transcription start site of a gene. **d** The number of hyper- and hypo-methylated TIMs in different genomic regions. **e** For samples where the true genome-wide methylation proportion was between 0.0 and 1.0 (red dots), the observed methylation proportion after capture and sequencing. For all box plots, the centerline of the box indicates the mean, the outer edges of the box indicate the upper and lower quartiles, and the whiskers indicate the maxima and minima of the distribution. Each dot indicates an individual

After collecting cfDNA, we turned to selecting methylation sites that are variable between tissues. In our previous work [10], we introduced the concept of tissue informative markers (TIMs) as a method to identify methylation sites that vary between tissues and cell types. Briefly, a TIM is a site that is either hyper- or hypo- methylated relative to the average methylation proportion of all other tissues at that site (Fig. 3a) (See Methods).

To find TIMs, we used reference WGBS methylomes that were obtained from public reference consortiums, ENCODE [32], BLUEPRINT [33], and IHEC. For this work, we focused on CpG sites as candidate TIMs, as most non-CpG sites are not methylated in adult tissues [46]. We selected approximately 250 TIMs for 18 tissues (Additional file 1: Table 5), which were prioritized based on recent deconvolution results from our previous work [10] and other recent works [22, 31] as core contributors to cfDNA to improve generalization to multiple disease contexts. These tissues included several hematopoietic cell types, organs, epithelium, and brain (Additional file 1: Table 5). We additionally include skeletal muscle due to its relevance to ALS. As reference datasets expand, the TIM selection process could be updated to include more tissues to further tailor the panel to disease-specific processes.

An important property of cfDNA is that their fragmentation patterns are non-random [47–49]. cfDNA observed in blood generally are fragments approximately 160 base pairs long [50], suggesting that cfDNA fragments are protected from degradation in the blood by the presence of tightly associated histone proteins. Since DNA methylation is enriched in nucleosome-bound regions, and cfDNA fragments predominantly originate from nucleosome-protected chromatin [49], cfDNA methylation profiles are biased toward methylated, heterochromatic regions. As a result, we chose to select a greater number of TIMs per tissue that were hypermethylated (Additional file 1: Table 5) (Fig. 3b).

After quality control (Methods), the final number of TIMS was 4,744. TIM sites were distributed throughout the genome (Additional file 1: Fig. S2a). Hypermethylated TIMs were closer, on average, to transcription start sites and CpG Islands than hypomethylated TIMs (Fig. 3c; Additional file 1: Fig. S2b). Since at a hypermethylated TIM, all other tissues are predominantly unmethylated, this observation is consistent with the role of DNA methylation in transcriptional repression and the presence of unmethylated CpGs at transcriptionally active loci [51]. Likewise, hypomethylated TIMs were more likely to be in intergenic and intronic regions (Fig. 3d), suggesting that in most tissues, these sites did not have a strong regulatory function. Together, this suggests that hypermethylated and hypomethylated TIMs offer complementary types of genomic information.

### Capture panel sequencing and validation

After designing the probes, we performed several validation experiments to ensure that probes could accurately profile the methylation state of the chosen TIMs. First, we used universal methylated DNA standards to create mixtures where the CpG sites were methylated 0, 25, 50, and 100% of the time. We captured the synthetic DNA mixtures with the probes and performed high-throughput sequencing. For each DNA mixture, we estimated the proportion of the time the captured CpG was methylated. We found that the observed methylation was highly concordant with the true methylation proportion (Fig. 3e), indicating that the probes were indeed quantifying the methylation accurately.

Next, to examine how the capture panel might perform in real-world cfDNA scenarios, we validated the capture panel using sheared genomic DNA from blood (*n* = 2), along with healthy cfDNA samples (*n* = 3). After performing cell-type deconvolution with CelFiE [10], we found that the sheared blood samples were estimated to be primarily composed of white blood cells as expected (Additional file 1: Fig. S3a). The majority of cfDNA from healthy controls was also estimated to be originating from neutrophils and lymphocytes, consistent with published research (Additional file 1: Fig. S3b) [52].

Lastly, we extracted cfDNA from a healthy control before and after vigorous exercise to examine the ability of the panel to measure tissue-specific changes in biological state. After capture and sequencing, we performed deconvolution of these two cfDNA samples. We found that cfDNA originating from neutrophils increased in the sample taken after exercise (Additional file 1: Fig. S3c), consistent with a recent report [53] studying the effect of exercise on cfDNA composition. Together, these experiments demonstrate that our approach for targeting TIMs can correctly capture the methylation state of cfDNA and measure relevant tissue of origin effects.

### CfDNA captured from ALS cases and controls

**Fig. 4:**
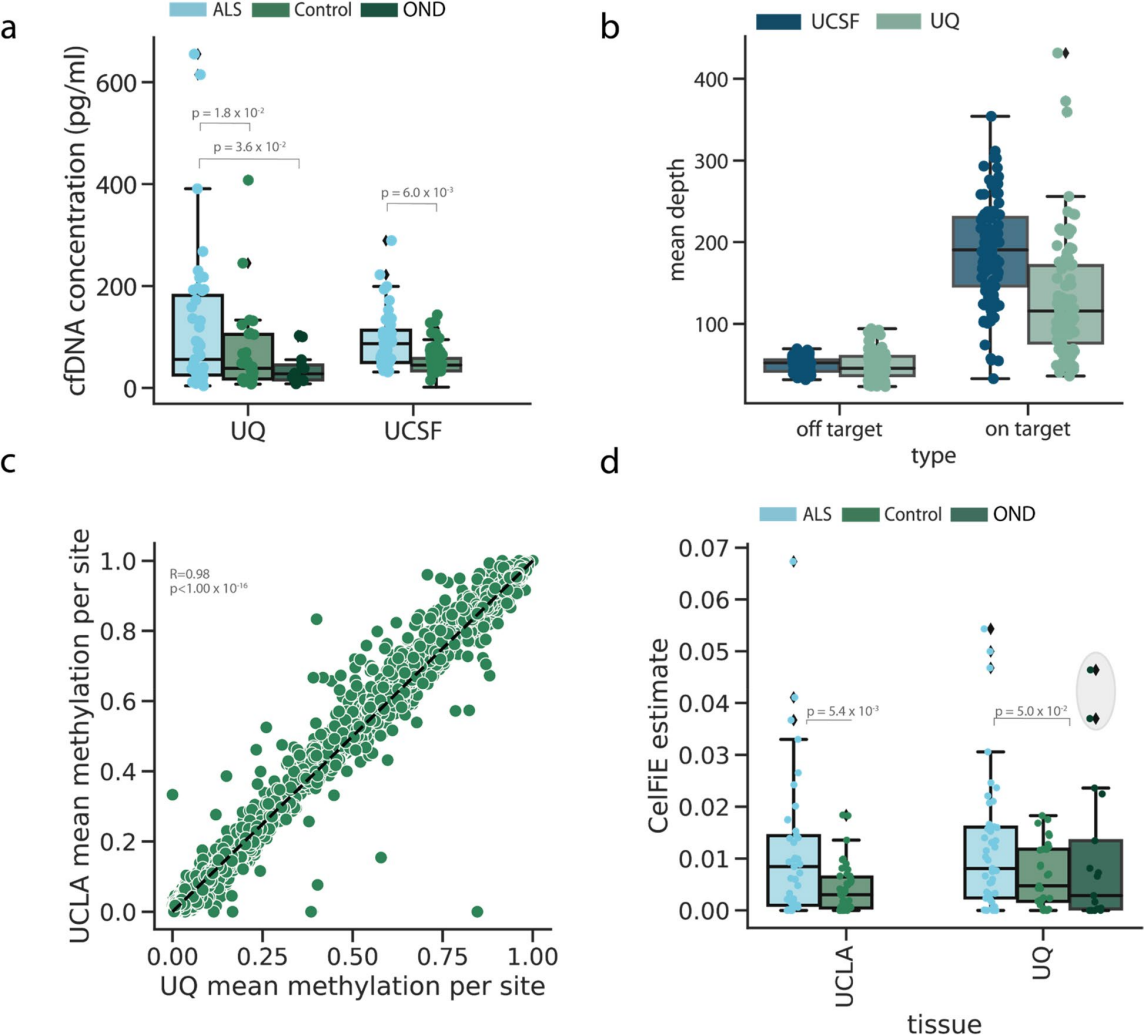
Capture panel performance on cfDNA data. **a** The starting cfDNA concentration of ALS patients and controls for each cohort, where each point represents one individual. **b** Coverage of the on-target and off-target CpG sites of each cohort, where each dot represents one sample. **c** Correlation between the UQ and UCSF methylation proportions at on-target sites. A single point represents a TIM. **d** The proportion of cfDNA from the controls and cases in each cohort that was estimated to originate from skeletal muscle. The gray-shaded circle indicates outlier control individuals. For all box plots, the centerline of the box indicates the mean, the outer edges of the box indicate the upper and lower quartiles, and the whiskers indicate the maxima and minima of the distribution. Each dot indicates an individual

We next turned to examining the cfDNA epigenome of our disease cohorts. cfDNA was extracted from the blood plasma of cases and controls from both UQ and UCSF patients. We first confirmed our previous finding [10] of an increased concentration of cfDNA in the plasma of ALS patients relative to controls (UQ mean ± SD: ALS = 124.84 ± 143.90 pg/mL, controls = 68.69 ± 84.47 pg/mL; UCSF mean ± SD: ALS = 95.92 ± 57.80 pg/mL, controls = 49.34 ± 27.49 pg/mL), a significant difference after correcting for age, sex, and SIRE (Fig. 4a) (logistic regression UQ: log odds ratio = 7.5 × 10^–3^, *p* = 1.8 × 10^–2^, UCSF: log odds ratio = 2.4 × 10^–2^, *p* = 6.0 × 10^–3^). Interestingly, cfDNA was also elevated in ALS patients relative to the OND controls (OND mean ± SD: 39.83 ± 29.39 pg/mL, logistic regression log odds ratio = 1.6 × 10^–2^, *p* = 3.6 × 10^–2^), which had overall low levels of cfDNA. This suggests that the cfDNA generative processes of apoptosis and necrosis might differ between ALS and other types of neurological diseases.

After quantifying the amount of cfDNA, we performed high-throughput methylation sequencing on the captured regions. Since bisulfite treatment can degrade the already low quantity of input DNA, cfDNA sequencing experiments are prone to high duplication [54]. To address this, we used unique molecular identifiers (UMIs) to deduplicate reads in both cohorts. In total, after sequencing and deduplication, the mean on-target coverage of UQ samples was 134 (SD: 166) reads per CpG and the mean on-target coverage of UCSF samples was 195 (SD: 229) reads per CpG. The large standard deviations are attributable to variability in sequencing coverage across sites. The average methylation proportion at TIM sites was highly correlated between the two cohorts (Pearson’s *R* = 0.98, *p* < 1.0 × 10^–16^) (Fig. 4c).

We noted that UCSF samples had a higher percentage of on-target reads (Additional file 1: Fig. S4), which likely contributed to differences in overall CpG read coverage. We also found that cfDNA starting concentration was a significant predictor of on target saturation after adjusting for total on target coverage (linear regression effect size = −1.7 × 10^–3^, *p* = 9.0 × 10^–3^) (Fig. S4d-e).

### Cell-type decomposition

Since TIMs were designed to be specific to a given tissue type, they can be used to estimate what tissues are contributing to the cfDNA in the context of neurodegeneration. To do this, we performed cfDNA cell-type decomposition with CelFiE [10]. CelFiE is a supervised decomposition algorithm that is designed to work with methylation read count data and missing or noisy reference data. As input, CelFiE takes the TIM read count data for each cfDNA sample and estimates the proportion of the cfDNA mixture originating from the tissues in the reference dataset, along with a specified number of unknown tissues.

**Fig. 5:**
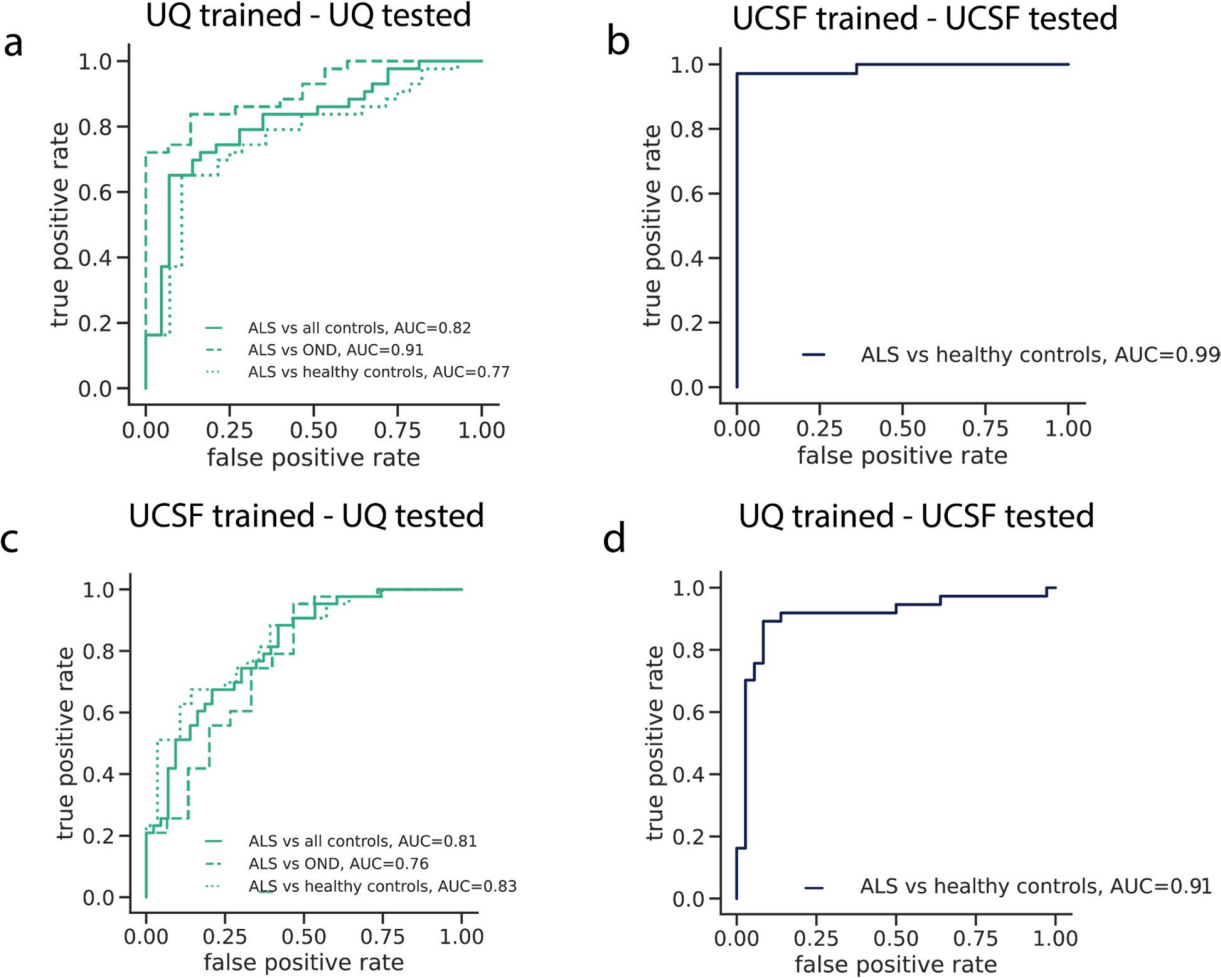
ALS disease classification with cfDNA epigenetic features. The false-positive rate versus true-positive rate for models trained and tested using CpG coverage, CpG methylation, and covariates as input features for **a** tenfold cross-validation within UQ samples, **b** tenfold cross-validation within UCSF samples, **c** trained on UCSF data and tested on UQ data, and **d** trained on UQ data and tested on UCSF data

We first ran CelFiE with three unknown components using the methylation status of the captured sites as input (Additional file 1: Fig. S5a). Then, we tested for differences in the estimated proportion of each tissue between the cases and controls in the UCSF cohort and examined those that were significantly different after multiple testing correction (FDR 10%). As with our prior ALS study [10], we observed elevated skeletal muscle in ALS patients. This was replicated in the UQ cohort (Mann–Whitney U test UCSF: *p* = 5.4 × 10^–3^, UQ: *p* = 5.0 × 10^–2^) (Fig. 5d). Increased cfDNA originating from skeletal muscle is consistent with muscle atrophy that occurs as part of their disease. Additionally, we observed increased cfDNA originating from heart ventricle, which was the specific heart tissue selected available in our ENCODE reference dataset, and may reflect cardiac degeneration more broadly (Additional file 1: Fig. S5b) (Mann–Whitney U test UCSF: *p* = 5.4 × 10^–3^, UQ: *p* = 4.7 × 10^–2^). This further illustrates the multisystem degeneration occurring in ALS.

Interestingly, we observed two UQ control samples with unusually high skeletal muscle components (an estimated 4.6% and 3.7% of their total cfDNA sample) (Fig. 5d). One sample was an OND control with frontotemporal dementia, a disease that has substantial genetic and clinical overlap with ALS [55]. The other sample was originally classified as a healthy control. However, after further investigation into their clinical records, this individual had both a parent and sibling with ALS. Genetic testing revealed that this individual also tested positive for a C9orf72 repeat expansion, which is the most common genetic cause of ALS [56], suggesting that the individual may be presymptomatic. Since the disease status of this patient was ambiguous, we reclassified them as OND.

### Classification of ALS disease status

While muscle degeneration is a hallmark of ALS, it is not specific enough to serve as a diagnostic tool. Therefore, to further characterize the relationship between alterations in the cfDNA epigenome and disease, we developed a tissue-agnostic algorithm that utilized information from all TIM DNA methylation profiles to predict whether a cfDNA sample was from an ALS patient or control. For these models, we did not consider PLS samples, but return to these samples in subsequent analyses. Further models integrated all CpG sites, both on and off target (See Methods).

We trained a penalized regression prediction model [35] in four contexts to explore the generalizability of the results across the independent cohorts. After parameter selection (see Methods), we found the best performing model across all four contexts was an elastic net regression. Model training began by using ten-fold cross-validation within each cohort. Then, the transferability of the models was assessed by training a model on one cohort and applying it to the other. Since only the UQ cohort had OND and healthy controls, we combined the controls for this analysis, although we later examined the ability of the model to discriminate between the different sample types. Model parameters, including the elastic net mixing parameter, were selected by using a cross-model selection and averaging procedure within the training set [35]. Non-penalized covariates included age at the time of cfDNA sampling, sex, SIRE, cfDNA concentration, and total cfDNA input. We evaluated model performance with area under the receiver operating characteristic curve (AUC) and by testing whether the predictions could significantly predict true case–control status using a logistic regression model that included covariates.

To best characterize the different types of information that TIMs can provide we explored two classes of features for the prediction model, the methylation proportion and the coverage of the TIMs normalized by total read count. Normalized coverage was included because cfDNA fragmentation is non-random; we therefore reasoned that CpG coverage may also be informative of disease status. In total, we trained models using CpG normalized coverage only, CpG methylation proportion only, and a combination of both as input features.

Overall, we found that tissue informative epigenetic features could significantly predict ALS case–control status in both cohorts (Fig. 5, Additional file 1: Fig. S6-7, Additional file 1: Table S6). The best-performing model incorporated both normalized TIM coverage, methylation features, and covariates (Fig. 5). Within cohorts, the ten-fold cross-validated AUC was 0.82 within the UQ cohort (logistic regression odds ratio = 2.34, *p* = 2.32⨉10^–7^) and the UCSF AUC was 0.99 (logistic regression odds ratio = 2.51, *p* < 2.0⨉10^–16^). The methylation and coverage only models that did not include covariate information significantly predicted disease status (Additional file 1: Fig. S6 and Additional file 1: Fig. S7), and were more predictive than models trained using only covariate information (Additional file 1: Fig. S8), suggesting the importance of the epigenetic features in predicting ALS. However, we noted that the covariate only performance was better in the UCSF samples (UCSF AUC = 0.70, UQ AUC = 0.50), potentially indicating that cohort differences could be enhancing UCSF model performance. Importantly, even though the model was not trained to distinguish between ALS cases and OND, the AUC was high for both UQ models (within UQ: AUC = 0.91, UCSF-UQ: AUC = 0.76).

Models trained within one cohort replicated between cohorts. We noted that the prediction performance was higher for the UQ-trained and UCSF-tested model (AUC = 0.91, logistic regression odds ratio = 1.92, *p* = 9.48 ⨉ 10^–5^) than the UCSF-trained model applied to the UQ samples (AUC = 0.81, logistic regression odds ratio = 2.46, *p* = 4.24⨉10^–4^). Differences in model performance between cohorts were likely driven by a combination of factors, including cohort heterogeneity and technical variation. One likely contributing factor was the lower on-target coverage in the UQ cohort (Fig. 4d, Additional file 1: Fig. S5). To test this, we randomly downsampled the number of reads in each UCSF cfDNA sample, which reduced effective on-target CpG coverage. Then, we re-ran the elastic net model within the UCSF cohort. We found that lower read coverage led to worse classification performance (Additional file 1: Fig. S9), suggesting that on-target CpG coverage is an important factor in prediction accuracy.

Importantly, the predictive performance of the elastic net models was stronger than using the CelFiE skeletal muscle estimate alone (Fig. 4d). Indeed, models trained without any skeletal muscle TIMs, did not have reduced performance relative to the full model (Additional file 1: Fig. S10), emphasizing the importance of combining information across tissue contributors.

We also noted that despite methylation proportion being the more common feature considered in epigenetic cfDNA studies, the models trained only using normalized CpG coverage also significantly predicted case–control status (Additional file 1: Table 6, Additional file 1: Fig. S6). In fact, there was very similar performance within the UCSF cross-validated model (AUC = 0.97) and the UQ cross-validated model (AUC = 0.84). This suggests that disease-relevant information is contained in simply the observation of a given CpG in cfDNA sequencing data, providing an additional layer of information over the CpG methylation state alone. This information may be lost in other low-cost epigenetic assays, like methylation arrays, that only return methylation proportion values.

Lastly, we considered the elastic net models that incorporated off-target CpGs (UCSF total number of CpGs = 32,314, UQ total number of CpGs = 49,238) as a proof of concept experiment. Since off-target CpGs were substantially lower coverage than the on-target sites, we performed less stringent CpG filtering to retain more off-target CpGs (see Methods). We found that the off-target models performed well (UCSF AUC = 0.86, UQ AUC = 0.76), even though this was a challenging setting as there were many more features than samples (Additional file 1: Fig. S11) and CpG coverage was lower. In future work, sites selected in these models could be chosen to refine TIM selection for capture panel development.

### Biological significance of prediction features

Next, we sought to understand how different tissue informative sites contributed to predicting disease. An advantage of using a regularized regression model like an elastic net is that the model performs feature selection and assigns a higher weight, or absolute β value, to features that contribute more to accurately predicting the outcome. Features that do not contribute to the prediction will have an absolute β value near zero. Thus, to examine the overall contribution of different types of TIMs in making model predictions, we obtained the absolute β value for each TIM from an elastic net model trained on the entire UQ and UCSF cohorts (Figs. 5c and d). Then we examined how these values related to different characteristics of the TIMs.

**Fig. 6:**
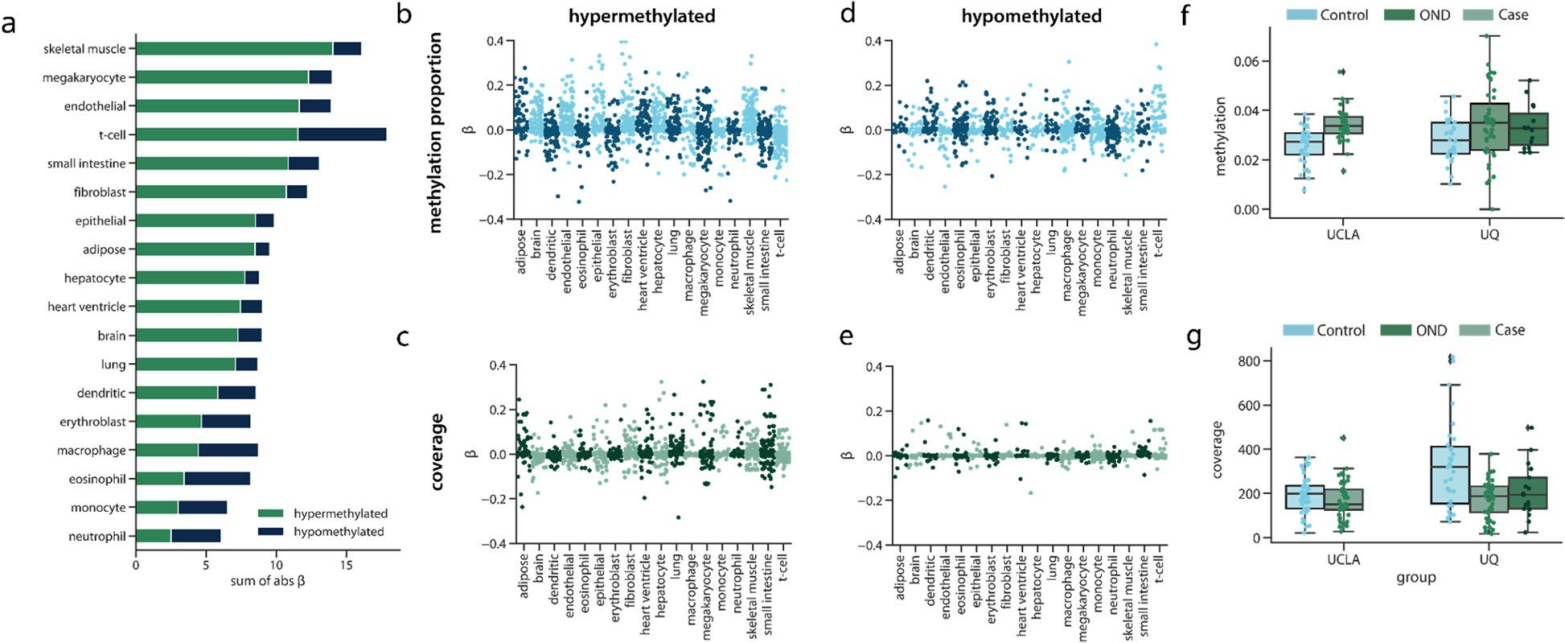
Features selected by the elastic net algorithm. **a** For each tissue, the TIMs were selected for, and for the type of TIM, the total absolute *β* value. A larger absolute *β* sum indicated that the feature type contributed more to model predictions. The *β* values for the **b** methylation proportion and **c** the read coverage of individual TIMs selected to be hypermethylated and the *β* values for the **d** methylation proportion and **e** read coverage of individual TIMs selected to be hypomethylated. **f** The methylation proportion of cases and controls for each cohort for a hypermethylated TIM in the *SHISA5* gene. **g** The read coverage of cases and controls for each cohort for a hypermethylated TIM located in the *XRCC6* gene. For all box plots, the centerline of the box indicates the mean, the outer edges of the box indicate the upper and lower quartiles, and the whiskers indicate the maxima and minima of the distribution. Each individual dot indicates a cfDNA sample

We first analyzed whether TIMs selected for a given tissue type were more important in making predictions. As expected, skeletal muscle TIMs were highly important in making model predictions, especially for TIMs that were hypermethylated in skeletal muscle (Fig. 6a). Despite the importance of skeletal muscle TIMs, we noted that TIMs for every tissue type contributed to the model predictions (Fig. 6). This again highlights the contribution of multiple tissues in neurodegeneration and the possibility of designing disease-specific biomarkers. For example, T-cell TIMs contributed to ALS disease prediction (Fig. 6a), indicating that cfDNA originating from immune cell types may be relevant in ALS disease.

Overall, there were differences in the importance of each class of TIM. Hypermethylated TIMs generally had higher absolute β values than hypomethylated TIMs (Fig. 6b-e), which could be related to our previous observation that hypermethylated TIMs were more likely to be in promoter or genic regions (Fig. 3). We also observed that there were differences in the distribution of absolute β values of methylation proportion and coverage features. For example, while methylation proportion features for fibroblast and epithelial cells had high absolute β values, coverage features for these tissues had relatively low absolute β values (Fig. 6b-c). Instead, the coverage of TIMs for small intestine and T-cells were high, but close to zero as methylation proportion features. Together, this could mean that including both methylation proportion and coverage of tissue informative sites is useful for learning about disease in the context of cfDNA.

We next examined individual TIMs as an avenue for examining and generating hypotheses about individual epigenetic biomarker candidates. TIMs with a non-zero absolute β value were chosen for association with ALS case–control status, along with covariates and correcting for cohort. Multiple test correction was employed using false discovery rate at 10%. One of the most important methylation proportion features was a hypermethylated TIM selected for epithelium. We observed significantly increased methylation in ALS cases for this TIM across cohorts (mean ± SD: ALS methylation proportion = 3.41 × 10^–2^ ± 1.17 × 10^–2^ control methylation proportion = 2.80 × 10^–2^ ± 8.52 × 10^–3^) (logistic regression odds ratio = 14.09, q-value = 8.06 × 10^–2^) suggesting that there was increased contribution from this gene in the cfDNA of ALS patients (Fig. 6f). This TIM was located in the promoter region of the *SHISA5* gene, which, along with *p53*, is involved in apoptosis [57]. Additionally, *SHISA5* was found to be over-expressed in the spinal cord of ALS patients [58].

We identified a similarly interesting hypermethylated TIM in the coverage features. While the TIM was selected for hepatocytes, it is in the *XRCC6* gene, which was highly expressed in many tissues in bulk RNA-seq from the Genotype-Tissue Expression (GTEx) Project [59]. Across cohorts, the TIM had significantly reduced coverage in ALS patients relative to controls (mean ± SD: ALS = 171.97 ± 86.40 reads, controls = 241.59 ± 151.12 reads) (logistic regression odds ratio = −42.25, q-value = 1.35 × 10^–2^) (Fig. 6g), and while it is difficult to infer directly from cfDNA alone, this result could suggest potential dysregulation of this gene in cases. *XRCC6* is involved in non-homologous end joining and DNA repair [60]. Disruption of non-homologous end joining has been previously linked to aging and ALS [61, 62].

### ALS disease phenotypes

To further explore the value of tissue-specific methylation sites as a potential biomarker, we developed models to predict ALS disease phenotypes. To do this, we trained three linear elastic net models to predict ALSFRS-R (*n* = 78), ALSFRS-R slope (*n* = 60), and FVC (*n* = 57) with ten-fold cross-validation. We hypothesized that high-weight features from the case–control analysis would also be associated with ALS phenotypes, and so, we chose the top 1000 coverage and top 1000 methylation features with the highest absolute β as input for the models. Since case-only numbers were relatively low in each cohort, we meta-analyzed the two cohorts, adding a non-penalized covariate for each cohort in the analysis, along with age, sex, cfDNA concentration, total cfDNA input quantity, and SIRE. To specifically evaluate the performance of cfDNA features over covariates, we separately trained an additional three models using only covariates.

**Fig. 7:**
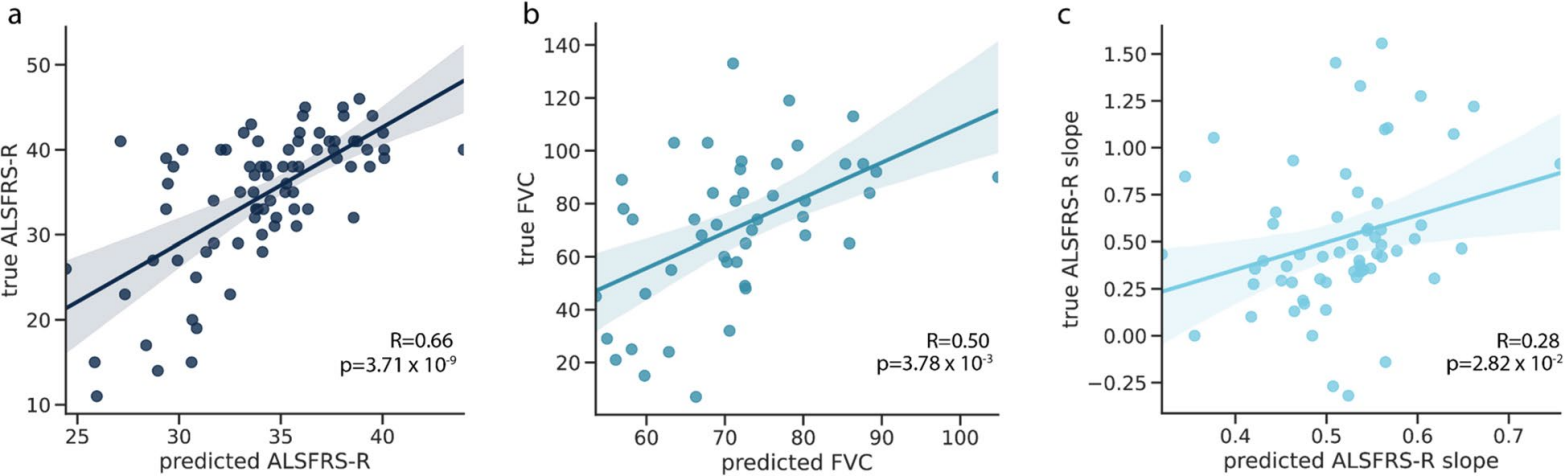
Predictive performance of cfDNA epigenetic features for ALS phenotypes. For a tenfold cross-validated model trained using cfDNA methylation proportion and coverage features of the predicted versus true **a** ALSFRS-R, **b** FVC, and **c** ALSFRS-R slope. Each point represents one ALS case

We found the models based on cfDNA epigenetic features significantly predicted ALSFRS-R (Fig. 7a) (Pearson’s R = 0.66, *p* = 3.71⨉10^–9^). This was significantly more predictive (*p* = 1.85⨉10^–5^) than the predictions from the model trained only on covariates (Pearson’s R = 0.46, *p* = 5.81⨉10^–5^). We found that the high predictive performance of the covariate-only model was largely attributed to cohort differences; within cohorts, the covariate model was not predictive (UQ: Pearson’s R = 8.48⨉10^–3^ *p* = 9.50⨉10^–1^, UCSF: Pearson’s R = 0.15, *p* = 3.80 × 10^–1^) but epigenetics remained predictive (UQ: Pearson’s R = 0.49, *p* = 8.52⨉10^–4^ UCSF: Pearson’s R = 0.54 *p* = 7.24⨉10^–4^).

We also found that the epigenetic models predicting FVC and ALSFRS-R slope were also significantly better than covariate-only models (FVC *p* = 2.67⨉10^–2^, ALSFRS-R slope *p* = 4.10⨉10^–2^), but more mild than the ALSFRS-R models (FVC Pearson’s R = 0.50, *p* = 3.78⨉10^–3^, ALSFRS-R slope Pearson’s R = 0.28, *p* = 2.81⨉10^–2^) (Fig. 7b-c). Like in the binary model, the important features were from a variety of tissues (Additional file 1: Fig. S12). TIMs for skeletal muscle contributed strongly to disease predictions for all three phenotypes. White blood cell related TIMs, however, were relatively less important. Interestingly, lung TIMs were amongst the top sites for all three models, which may be related to the decline of lung function as ALS progresses [63]. For FVC, endothelial cell TIMs were the top-rated feature, which is consistent with emerging literature on endothelial decline in ALS disease progression [64, 65]. Together, these results suggest that cfDNA epigenetic features are related to diverse biological processes underlying clinical traits used to measure ALS disease progression.

In complement to these analyses, we also assessed the hypothesis that epigenetic age determined via DNA methylation is associated with ALS disease status and progression [66, 67]. Using the same input TIMs, we trained a penalized regression model to predict age [68]. We found that using DNA methylation alone significantly predicted age (Pearson’s R = 0.60, *p* = 7.48 × 10^–17^) (Additional file 1: Fig. S13a). Then, we calculated age acceleration, which was defined as the residual between true and predicted age [69]. Larger age acceleration is a greater difference between actual age and epigenetic age, which has been found to be predictive of a variety of diseases. We observed that ALS cases had greater age acceleration than controls, (logistic regression odds ratio = 1.01, *p*-value = 3.1 × 10^–2^), consistent with past research (Additional file 1: Fig. S13b) [66]. We noted that age acceleration in ALS patients was more advanced in the UCSF cohort (mean ± SD: ALS = 2.12 ± 9.89, Controls = −0.97 ± 9.31) than in the UQ samples (mean ± SD: ALS = −0.097 ± 7.83, Controls = −3.48 ± 8.05). Age acceleration also was significant in predicting ALSFRS-R slope after controlling for covariates including actual age (linear regression β = 0.054, *p* = 1.7 × 10^–2^), suggesting that epigenetic age acceleration measured via cfDNA may be useful in understanding ALS disease progression [70, 71].

Lastly, we studied whether the same cfDNA epigenetic features that were associated with ALS disease phenotypes could differentiate between ALS and PLS cases. Due to the small sample number of PLS cases (*n* = 15), we again combined the two cohorts and fit using fivefold cross-validation with a non-penalized parameter for cohort. Although the analysis was underpowered, we observed a statistically significant difference between model predictions for ALS and PLS cases (AUC = 0.74, linear regression effect size = 36.61, *p* = 1.9⨉10^–2^).

## Discussion

Here, we presented a scalable cfDNA capture protocol that measures the methylation status of disease and tissue relevant CpG sites. We applied this capture technology to two independent cohorts of ALS patients and age-matched controls and examined the correlation with ALS disease status and progression. We then integrated both the read coverage and methylation proportion of the targeted sites in a machine-learning model. This model significantly discriminated between ALS patients and controls in two independent cohorts, including those with a variety of other neurological diseases. Together, our results suggest that a capture approach targeting tissue informative DNA methylation markers has value in quantitative biomarker development. In particular, cfDNA may serve as a potential biomarker for ALS in neurology specialty settings, where individuals are being assessed for symptoms consistent with a neurodegenerative disorder. Estimates suggest that ALS prevalence among individuals presenting with such symptoms could be 5% or higher, depending on referral patterns and patient demographics. At this prevalence, a cfDNA test with 90% sensitivity and specificity, would yield a positive predictive value of ~ 32%. While encouraging, further work is needed to rigorously calibrate and validate cfDNA as a biomarker for ALS before widespread clinical adoption.

A key strength of using methylation markers informative of a broad variety of tissues is that it facilitates a comprehensive picture of a patient’s biological state and is not limited to a specific tissue or context. For example, neurofilament light chain is an exciting biomarker candidate for ALS [72–74]. However, neurofilament light chain also is elevated in other neurodegenerative diseases, which might limit its specificity for some applications [75]. By capturing and quantifying methylation levels at multiple tissue-informative CpG sites simultaneously, the panel has the potential to also learn about biological processes occurring in ALS outside of neurodegeneration. For example, we observed a strong signal originating from skeletal muscle. While skeletal muscle degeneration may be more easily observed in plasma cfDNA than neural tissue, the importance of skeletal muscle aligns with recent reports suggesting it may be a primary driver of disease mechanisms and heterogeneity [76]. Several recent works have also nominated skeletal muscle degeneration as a potential biomarker for ALS [77, 78].

Additionally, cfDNA is well-suited to measuring inflammation [8, 52], which has been of recent interest in ALS pathophysiology [79, 80]. While other groups have identified a contribution of monocytes and macrophages to ALS disease progression [81, 82], we do not identify that here. Instead, we identify a contribution of T-cells, which may be consistent with recent research on the contribution of T-cells to ALS disease [83–85]. However, our ability to detect inflammatory signatures may be limited by focusing on healthy white blood cells in TIM selection and by the rates of DNA being shed into the bloodstream [86]. As new reference panels develop [21], additional tissues and cell types could be incorporated into the TIM selection workflow to increase sensitivity in ALS and to improve generalizability other disease contexts.

We observed differential performance between the UQ and UCSF cohorts. One explanation for this performance difference is heterogeneity in the composition of the UCSF and UQ cohorts. This is underscored by the superior predictive performance of the UCSF covariate-only model, which could result in potential cohort-specific confounding. Future work can focus on expanding ALS disease cohorts and collect detailed clinical records to identify potential confounders. Additionally, DNA methylation is highly correlated with sample characteristics and the environment. For example, we identified a significant association between DNA methylation age and ALS. Differences in the fundamental underlying methylation of the cohorts could contribute to differential performance.

Another explanation may be attributed to differences in sequencing depth. The UCSF cohort had higher on-target CpG coverage. Additional coverage may reduce noise, especially in analyses utilizing methylation proportion. In some cases, the overall coverage is limited by the total amount of cfDNA available as input to the sequencing assay. This could be improved by recent high-throughput extraction technologies with the ability to increase cfDNA yield from a plasma sample [87, 88].

Model performance also may be affected by the slight differences in ALS patient characteristics between the cohorts. For example, the UCSF cohort had patients with lower ALSFRS-R scores and whose advanced condition may be easier to detect in cfDNA. ALS is also an extremely heterogeneous disease [89], which can make designing biomarkers that generalize across patient populations difficult. It is also important to note that both cohorts were of majority European ancestry. Further exploration of how epigenetic cfDNA profiles differ between diverse subtypes of patients or change longitudinally as patients progress is now needed.

This study also only examined the performance of tissue informative markers in characterizing ALS. Since initiating these studies, other proposed blood-based biomarkers for ALS, like neurofilaments [75, 90, 91], proteomics [92], or miRNA [13] have demonstrated promise and future studies will need to benchmark with at least one of these. Previous studies have also illustrated the benefit of combining different types of biomarkers to enhance predictive performance. Future work on cfDNA biomarker development in ALS could assay multiple biofluids simultaneously and include a range of cohorts (i.e. asymptomatic gene-positive carriers for diagnosis, multi-ancestry, neurological conditions presenting with weakness). Integration of these multiple measurements, along with information about existing patient genetic liability, would robustly test its potential context of use and may improve disease prediction models.

Lastly, there are numerous avenues for improving algorithms associated with the approach outlined here. While methylation capture arrays allow for a more cost-effective and focused analysis over relevant CpG sites, targeted capture also limits the coverage of the genome. This has the potential to miss important methylation changes occurring outside the targeted regions. Additionally, since we relied on published tissue methylation data sets that are low coverage and inherently noisy, TIM selection might be affected. Marker selection and overall algorithm performance might be improved by better, high-coverage reference data. Furthermore, the TIM algorithm could be improved by considering regions, instead of single CpGs, or designing algorithms to detect regions of correlated methylation, as in the differential methylation literature [93]. Reference panel design for cfDNA applications is a robust area of current research, which can impact the accuracy of cfDNA results. Incorporating new large-scale reference datasets presents an opportunity for improving the resolution of cfDNA tissue-of-origin research, both in ALS and in other disease contexts. Finally, single-molecule [94] and nonlinear models [48] have shown recent promise in the analysis of cfDNA profiles.

## Conclusions

Overall, the design of the cell-free DNA methylation capture panel and related prediction algorithms presented in this study represents an advancement in the study of cfDNA in diverse disease contexts, especially neurodegeneration. These findings establish epigenetic cfDNA analysis as a promising quantitative tool for ALS diagnosis and monitoring, while offering insights into disease mechanisms through tissue-specific degeneration patterns.

## Supplementary Information

Additional file 1: Supplementary figures: Figure S1: *Cohort demographic characteristics*. For the UQ and UCSF cohorts, (a) the distribution of the age of the cases and controls, (b) the percentage of the cohorts that are female, and the percentage of the (c) ALS cases and (d) controls that identify as five different racial/ethnic categories. Figure S2: *Properties of captured TIMs* (a) The number of TIMs selected per chromosome and (b) for the two types of TIMs, the distribution of distances between a TIM and a CpG island. Figure S3: *Deconvolution of validation data*. The CelFiE estimates (a) for sheared genomic DNA (*n* = 2) samples taken from blood and (b) healthy cfDNA (*n* = 3). (c)For cfDNA taken from one individual before and after exercise, the proportion of cfDNA estimated to be originating from neutrophils. Figure S4: *On target percentage*. The percentage of reads that were on-target (a) before deduplication and (b) after deduplication. For each cohort, (c) the percentage of the total mapped starting reads before deduplication that remained after deduplication. The on-target saturation, defined as 1-(median depth on target after deduplication/median depth on target before deduplication) for (d) the UCSF cohort and (e) the UQ cohort. Figure S5: *Cell-type decomposition estimates.* (a) The proportion of cfDNA estimated by CelFiE to originate from each tissue for each sample type. (b) The CelFiE estimate of heart ventricle for each sample type in each cohort. Figure S6: *ALS disease classification using CpG coverage.* The false positive rate versus true positive rate for models trained and tested using only CpG coverage as input features and no covariate information for (a) ten fold cross validation within UQ samples (b) ten fold cross validation within UCSF samples (c) trained on UCSF data and tested on UQ data, and (d) trained on UQ data and tested on UCSF data. Figure S7: *ALS disease classification using CpG methylation.* The false positive rate versus true positive rate for models trained and tested using only CpG methylation proportion as input features and no covariate information for (a) ten fold cross validation within UQ samples (b) ten fold cross validation within UCSF samples (c) trained on UCSF data and tested on UQ data, and (d) trained on UQ data and tested on UCSF data. Figure S8: *ALS disease classification using only covariate information.* The false positive rate versus true positive rate for models trained and tested using only covariate information (age, sex, and SIRE) as input features for (a) ten fold cross validation within UQ samples (b) ten fold cross validation within UCSF samples (c) trained on UCSF data and tested on UQ data, and (d) trained on UQ data and tested on UCSF data. Figure S9: *The relationship between read coverage and predictive performance.* For UCSF cfDNA samples, the total number of reads was randomly downsampled to reduce overall on-target CpG coverage relative to the actual UCSF read coverage. The downsampled samples were then used as input for elastic net models trained using tenfold cross validation to predict case–control status in the UCSF cohort and the AUC was recorded. The within-cohort UQ AUC is indicated by a red X. Figure S10: *ALS disease classification without skeletal muscle TIMS.* The false positive rate versus true positive rate for models trained and tested using cfDNA CpG methylation, CpG coverage, and covariate information (age, sex, SIRE, starting cfDNA concentration, and total cfDNA input) for all TIMs besides those chosen for skeletal muscle as input features for (a) ten fold cross validation within UQ samples (b) ten fold cross validation within UCSF samples (c) trained on UCSF data and tested on UQ data, and (d) trained on UQ data and tested on UCSF data. Figure S11: *ALS disease classification with off-target CpGs.* The false positive rate versus true positive rate for models trained and tested using off target cfDNA CpG methylation trained and tested used (a) ten fold cross validation within UCSF samples (b) ten fold cross validation within UQ samples. Figure S12: *Weight of features predicting ALS phenotypes.* For each tissue the TIMs were selected for, and for the type of TIM, the total absolute β value for (a) ALSFRS-R (b) FVC and (c) ALSFRS-R slope phenotypes. A larger absolute β sum indicated that the feature type contributed more to model predictions. Figure S13: *Epigenetic age acceleration in ALS.* (a) The association between predicted age via DNA methylation and the true age of participants across the UQ and UCSF cohorts. (b) The age acceleration of ALS patients, healthy controls, and OND patients. Supplementary tables: Table S1: *Clinical characteristics of ALS patients.* The clinical and demographic characteristics per cohort. The number of total patients is shown, and the number of female patients is shown in parentheses. Table S2: *Familial disease status.* For the ALS patients, the number that was reported as sporadic, familial, or unknown. Table S3: *Number of patients with alterations in genes associated with ALS.* For the UQ cohort, where some patients had test results for ALS or motor neuron associated genes available, the number of patients that are positive or negative for a specific gene. Note that patients may be positive for more than one gene. For some patients in the UQ cohort, and all patients in the UCSF cohort, genetic information was unavailable. Table S4: *Other neurological disease patients.* For each of the controls with other neurological diseases in the UQ cohort, the type of neurological disease (if known) and the number of patients with that disease. Table S5: *TIM selection design.* Per tissue selected for capture, the number of hypermethylated TIMs selected, the number of hypomethylated TIMs selected, and the total number of final TIMs selected for capture. Table S6: *Binary prediction model performance.* The AUC of predicting ALS vs all control samples for four models trained either within a cohort or trained in one cohort and tested on the remaining cohort. Models were trained with either only CpG coverage as input features, only CpG methylation, or both. Table S7: *WGBS reference data accession information.* For each of the WGBS reference data sets used for TIM selection and for deconvolution, the identifier and original source of the reference. Table S8: *Parameters for penalized regression models.* For each regression model, the alpha parameter and lambda value selected by the CMSA procedure in BigStatsR.

## Data Availability

Scripts to replicate analyses can be found at https://github.com/christacaggiano/cfdna-tims [37]. Code to calculate original TIMs and perform CelFiE can be found at https://github.com/christacaggiano/celfie [24]. Fastq files, bed files, and associated metadata for the samples are freely available at NCBI GEO GSE307705 (https://www.ncbi.nlm.nih.gov/geo/query/acc.cgi?acc=GSE307705). Tissue and cell-type WGBS data is freely available from the ENCODE Project (https://www.encodeproject.org/) [95], the BLUEPRINT Epigenome Project (https://projects.ensembl.org/blueprint/) [33] data access portals. The Canadian Epigenetics, Environment, and Health Research Consortium data was accessed from the IHEC data portal (https://epigenomesportal.ca/ihec/)0.20.
