# Supplementary material for: Epigenetic profiles of tissue informative CpGs inform ALS disease status and progression
Source: Genome Med. 2025 Oct 16;17:115. doi: 10.1186/s13073-025-01542-5 (PMC12529837; doi:10.1186/s13073-025-01542-5)
Supplement: Supplementary file 1 — Additional file 1: Supplementary figures: Figure S1: Cohort demographic characteristics. For the UQ and UCSF cohorts, (a) the distribution of the age of the cases and controls, (b) the percentage of the cohorts that are female, and the percentage of the (c) ALS cases and (d) controls that identify as five different racial/ethnic categories. Figure S2: Properties of captured TIMs (a) The number of TIMs selected per chromosome and (b) for the two types of TIMs, the distribution of distances between a TIM and a CpG island. Figure S3: Deconvolution of validation data. The CelFiE estimates (a) for sheared genomic DNA (n = 2) samples taken from blood and (b) healthy cfDNA (n = 3). (c)For cfDNA taken from one individual before and after exercise, the proportion of cfDNA estimated to be originating from neutrophils. Figure S4: On target percentage. The percentage of reads that were on-target (a) before deduplication and (b) after deduplication. For each cohort, (c) the percentage of the total mapped starting reads before deduplication that remained after deduplication. The on-target saturation, defined as 1-(median depth on target after deduplication/median depth on target before deduplication) for (d) the UCSF cohort and (e) the UQ cohort. Figure S5: Cell-type decomposition estimates. (a) The proportion of cfDNA estimated by CelFiE to originate from each tissue for each sample type. (b) The CelFiE estimate of heart ventricle for each sample type in each cohort. Figure S6: ALS disease classification using CpG coverage. The false positive rate versus true positive rate for models trained and tested using only CpG coverage as input features and no covariate information for (a) ten fold cross validation within UQ samples (b) ten fold cross validation within UCSF samples (c) trained on UCSF data and tested on UQ data, and (d) trained on UQ data and tested on UCSF data. Figure S7: ALS disease classification using CpG methylation. The false positive rate versus true positive rate [file 13073_2025_1542_MOESM1_ESM.docx]

## Supplementary Tables

|  | **UCSF** | **UQ** |
| --- | --- | --- |
| **Number of ALS patients** | 41 (female=20) | 43 (female=14) |
| **Number of PLS patients** | 9 (female=1) | 5 (female=2) |
| **Number of healthy controls** | 45 (female=27) | 31 (female=21) |
| **Number of asymptomatic gene carriers** | 0 | 2 (female = 1) |
| **Number of OND controls** | 0 | 15 (female = 8) |
| **Age** (mean ± SD) | 62.3 ± 11.9 | 61.0 ± 9.4 |
| **Sex** (percentage female) | 54.7% | 39.1% |
| **ALSFRS-R** (mean ± SD) | 31.5 ± 9.3 | 32.4 ± 6.6 |
| **ALSFRS-R slope** (mean ± SD) | 0.7 ± 0.8 | 0.5 ± 1.1 |
| **Age of onset** (mean ± SD) | 61.3 ± 12.6 | 58.6 ± 8.5 |
| **Days since disease onset** (mean ± SD) | 1824.9 ± 2077.9 | 938.1 ± 847.8 |

#### **Table S1: *Clinical characteristics of ALS patients.*** The clinical and demographic characteristics per cohort. The number of total patients is shown, and the number of female patients is shown in parentheses.

| **Cohort** | **unknown** | **sporadic** | **familial** |
| --- | --- | --- | --- |
| **UCSF** | 46 (female=21) | 0 (female=0) | 2 (n=1) |
| **UQ** | 3 (female=1) | 39 (female=11) | 6 (female=3) |

#### **Table S2: *Familial disease status.*** For the ALS patients, the number that was reported as sporadic, familial, or unknown.

|  | **Status** | **Genetic status unknown** | ***c9orf72***  **positive** | ***SOD1***  **positive** | **Gene negative** |
| --- | --- | --- | --- | --- | --- |
| **UQ** | ALS/PLS | 22 (female=9) | 2 (female=0) | 1 (female=0) | 14 (female=6) |
|  | Control | 46 (female=29) | 2 (female=1) | 0 (female=0) | 0 (female=0) |
| **UCSF** | ALS/PLS | 50 (female=21) | 0 (female=0) | 0 (female=0) | 0 (female=0) |
|  | Control | 45 (female=27) | 0 (female=0) | 0 (female=0) | 0 (female=0) |

#### **Table S3: *Number of patients with alterations in genes associated with ALS.*** For the UQ cohort, where some patients had test results for ALS or motor neuron associated genes available, the number of patients that are positive or negative for a specific gene. Note that patients may be positive for more than one gene. For some patients in the UQ cohort, and all patients in the UCSF cohort, genetic information was unavailable.

| **Disease Type** | **UQ OND Controls** |
| --- | --- |
| Alzheimer’s disease | 5 |
| Progressive supranuclear palsy | 3 |
| Frontotemporal degeneration | 2 |
| Other neurological disease | 1 |
| Parkinson’s dementia (Lewy Body disease) | 1 |
| Corticobasal syndrome | 1 |
| Semantic dementia | 1 |
| Dementia with Lewy bodies | 1 |

#### **Table S4: *Other neurological disease patients.*** For each of the controls with other neurological diseases in the UQ cohort, the type of neurological disease (if known) and the number of patients with that disease.

####

| **Tissue** | **Hypermethylated** | **Hypomethylated** | **Total** |
| --- | --- | --- | --- |
| **Adipose** | 208 | 42 | 250 |
| **Brain** | 200 | 50 | 250 |
| **Dendritic cell** | 179 | 71 | 250 |
| **Endothelial cell** | 200 | 50 | 250 |
| **Eosinophil** | 83 | 167 | 250 |
| **Erythroblast** | 134 | 116 | 250 |
| **Fibroblast** | 200 | 50 | 250 |
| **Heart left ventricle** | 204 | 46 | 250 |
| **Hepatocyte** | 200 | 50 | 250 |
| **Lung left lobe** | 200 | 45 | 245 |
| **Macrophage** | 86 | 164 | 250 |
| **Epithelial** | 200 | 50 | 250 |
| **Megakaryocyte** | 184 | 66 | 250 |
| **Monocyte** | 72 | 164 | 236 |
| **Neutrophil** | 66 | 184 | 250 |
| **Skeletal muscle** | 200 | 50 | 250 |
| **Small intestine** | 200 | 50 | 250 |
| **T-cell** | 290 | 223 | 513 |

#### **Table S5:** ***TIM selection design.*** Per tissue selected for capture, the number of hypermethylated TIMs selected, the number of hypomethylated TIMs selected, and the total number of final TIMs selected for capture.

##

##

| **Training** | **Test** | **Input Features** | **AUC (ALS vs all controls)** | **Odds Ratio** | **P-value** |
| --- | --- | --- | --- | --- | --- |
| UCSF | UCSF | Coverage only | 0.97 | 2.53 | <2.0 ⨉ 10^-16^ |
| UCSF | UCSF | Methylation proportion only | 0.99 | 2.54 | <2.0 ⨉ 10^-16^ |
| UCSF | UCSF | Both | 0.99 | 2.51 | <2.0 ⨉ 10^-16^ |
| UQ | UCSF | Coverage only | 0.74 | 1.12 | 6.40 ⨉ 10^-1^ |
| UQ | UCSF | Methylation proportion only | 0.91 | 2.15 | 8.54 ⨉ 10^-6^ |
| UQ | UCSF | Both | 0.91 | 1.92 | 9.48 ⨉ 10^-5^ |
| UCSF | UQ | Coverage only | 0.71 | 25.11 | 1.17 ⨉ 10^-1^ |
| UCSF | UQ | Methylation proportion only | 0.79 | 2.08 | 2.43 ⨉ 10^-4^ |
| UCSF | UQ | Both | 0.81 | 2.46 | 4.24 ⨉ 10^-4^ |
| UQ | UQ | Coverage only | 0.84 | 2.90 | 3.07 ⨉ 10^-8^ |
| UQ | UQ | Methylation proportion only | 0.80 | 2.45 | 2.67 ⨉ 10^-6^ |
| UQ | UQ | Both | 0.82 | 2.34 | 2.32 ⨉ 10^-7^ |

##

#### **Table S6:** ***Binary prediction model performance.*** The AUC of predicting ALS vs all control samples for four models trained either within a cohort or trained in one cohort and tested on the remaining cohort. Models were trained with either only CpG coverage as input features, only CpG methylation, or both.

##

##

| Tissue | Full name | Sample ID | Source |
| --- | --- | --- | --- |
| Adipose | adipose | ENCFF318AMC, ENCFF477GKI | ENCODE |
| Brain | brain | CEMT0022, CEMT0023 | CEEHRC |
| Dendritic cell | conventional dendritic cell | S00CP651, S00D71 | BLUEPRINT |
| Endothelial cell | endothelial cell of umbilical vein (resting) | S00DCS, S00BJM | BLUEPRINT |
| Eosinophil | mature eosinophil | S00V65, S006XE | BLUEPRINT |
| Erythroblast | erythroblast | S002S3, S002R5 | BLUEPRINT |
| Fibroblast | fibroblast | ENCFF714SUO, ENCFF752NXS | ENCODE |
| Heart ventricle | heart left ventricle | ENCBS663RCE, ENCBS457GBV | ENCODE |
| Hepatocyte | HepG2 | ENCFF847OWL, ENCFF064GJQ | ENCODE |
| Lung left lobe | upper left lobe of lung | ENCBS482XWK, ENCBS621MSH | ENCODE |
| Macrophage | macrophage | S0022I, S00390 | BLUEPRINT |
| Epithelial | mammary epithelial | CEMT0009, CEMT0008 | CEEHRC |
| Megakaryocyte | megakaryocyte | MK_BM_I21, MK_BM_I22 | BLUEPRINT |
| Monocyte | monocyte | Primary_monocytes_Donor_G, Primary_monocytes_Donor_D | BLUEPRINT |
| Neutrophil | mature neutrophil | C0010K, C000S5 | BLUEPRINT |
| Skeletal muscle | Skeletal muscle myoblast | ENCFF774GXJ | ENCODE |
| Small intestine | small intestine | ENCFF266NGW, ENCFF122LEF | ENCODE |
| T-cell | CD4-positive, alpha-beta T cell | S007G7, S007DD | BLUEPRINT |

#### **Table S7: *WGBS reference data accession information.*** For each of the WGBS reference data sets used for TIM selection and for deconvolution, the identifier and original source of the reference.

##

##

| Training | Test | Task | Alpha | Lambda |
| --- | --- | --- | --- | --- |
| UCSF | UQ | ALS status | 0.0001 | 11.94 |
| UQ | UCSF | ALS status | 0.0001 | 33.90 |
| UCSF | UCSF | ALS status | 0.01 | 11.56 |
| UQ | UQ | ALS status | 0.0001 | 24.10 |
| All | All | FVC | 0.2 | 81.72 |
| All | All | ALSFRS slope | 0.0001 | 1793 |
| All | All | ALSFRS-R | 0.1 | 39.67 |

##

#### **Table S8: *Parameters for penalized regression models.*** For each regression model, the alpha parameter and lambda value selected by the CMSA procedure in BigStatsR.

##

## Supplementary Figures

##
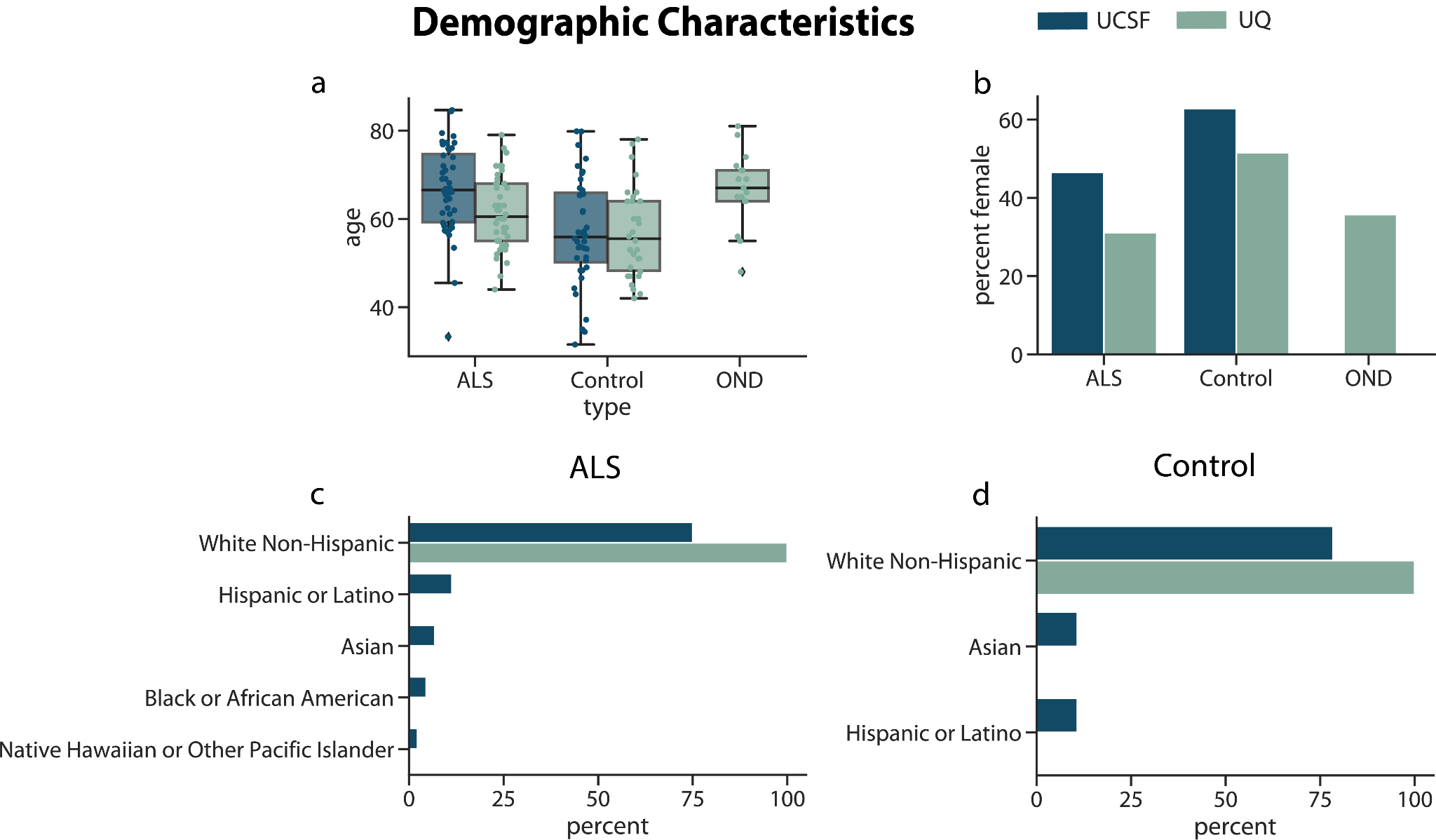


##### **Figure S1:** ***Cohort demographic characteristics*.** For the UQ and UCSF cohorts, (a) the distribution of the age of the cases and controls, (b) the percentage of the cohorts that are female, and the percentage of the (c) ALS cases and (d) controls that identify as five different racial/ethnic categories.

#####

#####

#####


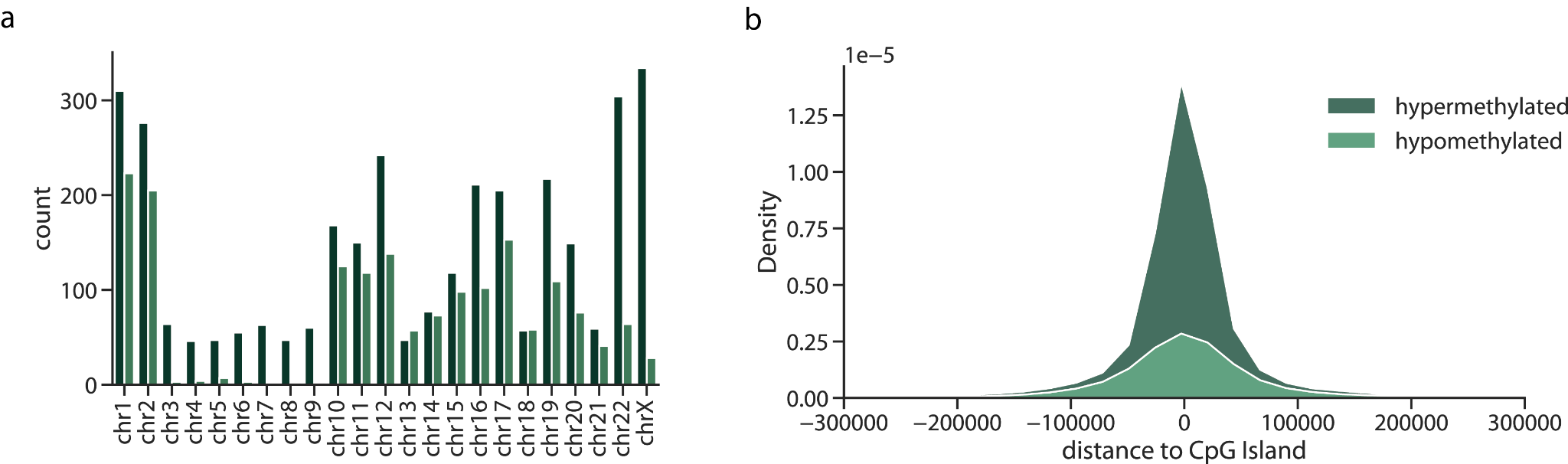


##### **Figure S2:** ***Properties of captured TIMs* (a)** The number of TIMs selected per chromosome and (b) for the two types of TIMs, the distribution of distances between a TIM and a CpG island.

##### **
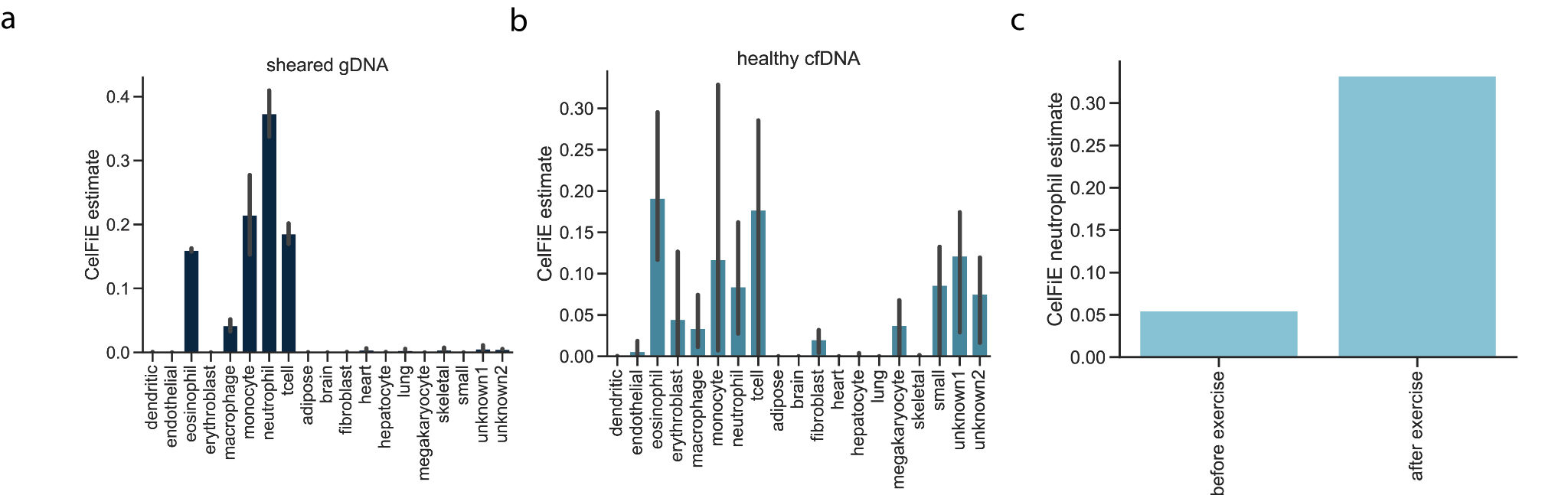
**

##### **Figure S3:** ***Deconvolution of validation data*.** The CelFiE estimates **(a)** for sheared genomic DNA (n=2) samples taken from blood and **(b)** healthy cfDNA (n=3). **(c)**For cfDNA taken from one individual before and after exercise, the proportion of cfDNA estimated to be originating from neutrophils.

##### **
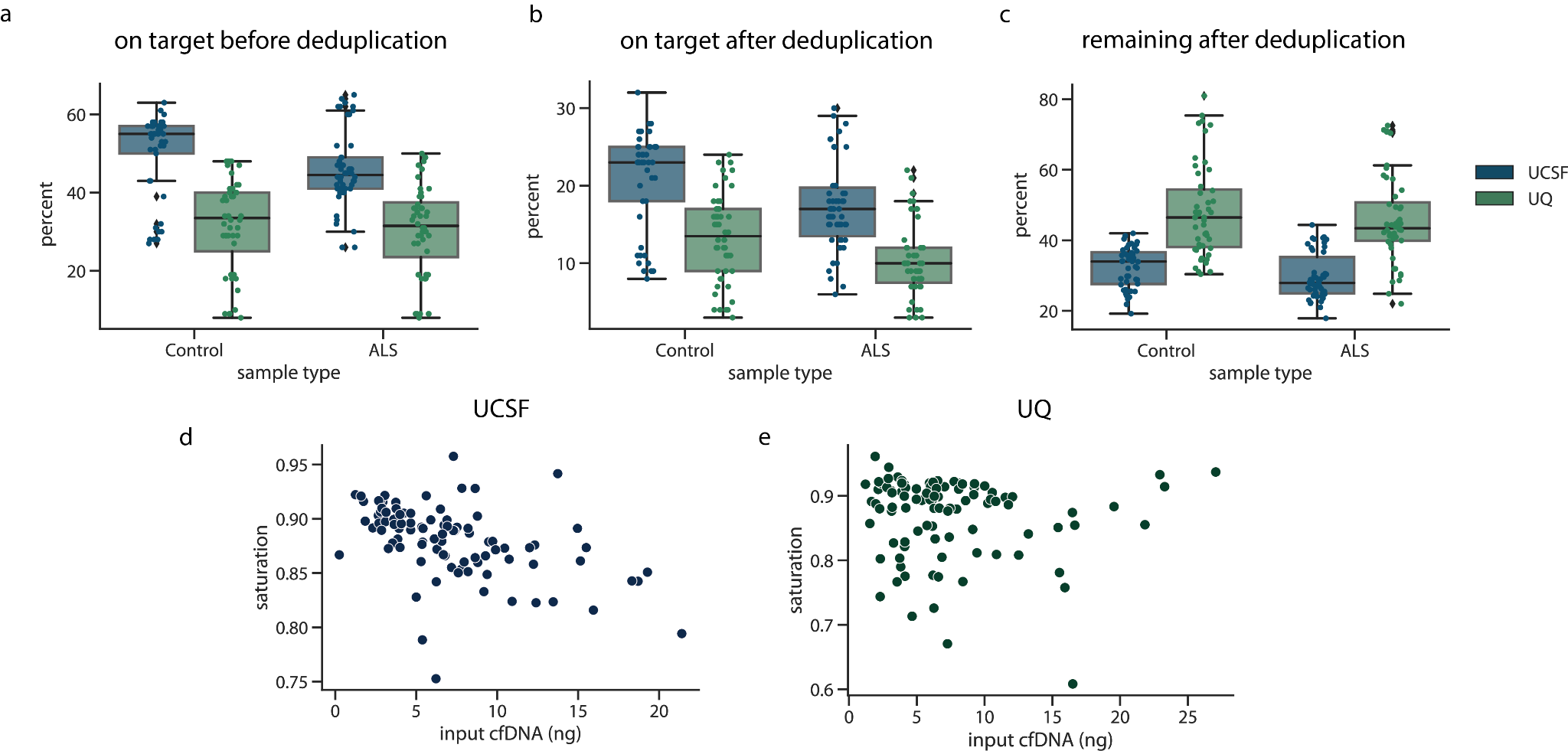
**

##### **Figure S4:** ***On target percentage*.** The percentage of reads that were on-target (**a)** before deduplication and (**b)** after deduplication. For each cohort, (**c)** the percentage of the total mapped starting reads before deduplication that remained after deduplication. The on-target saturation, defined as 1-(median depth on target after deduplication / median depth on target before deduplication) for (d) the UCSF cohort and (e) the UQ cohort.

##### **
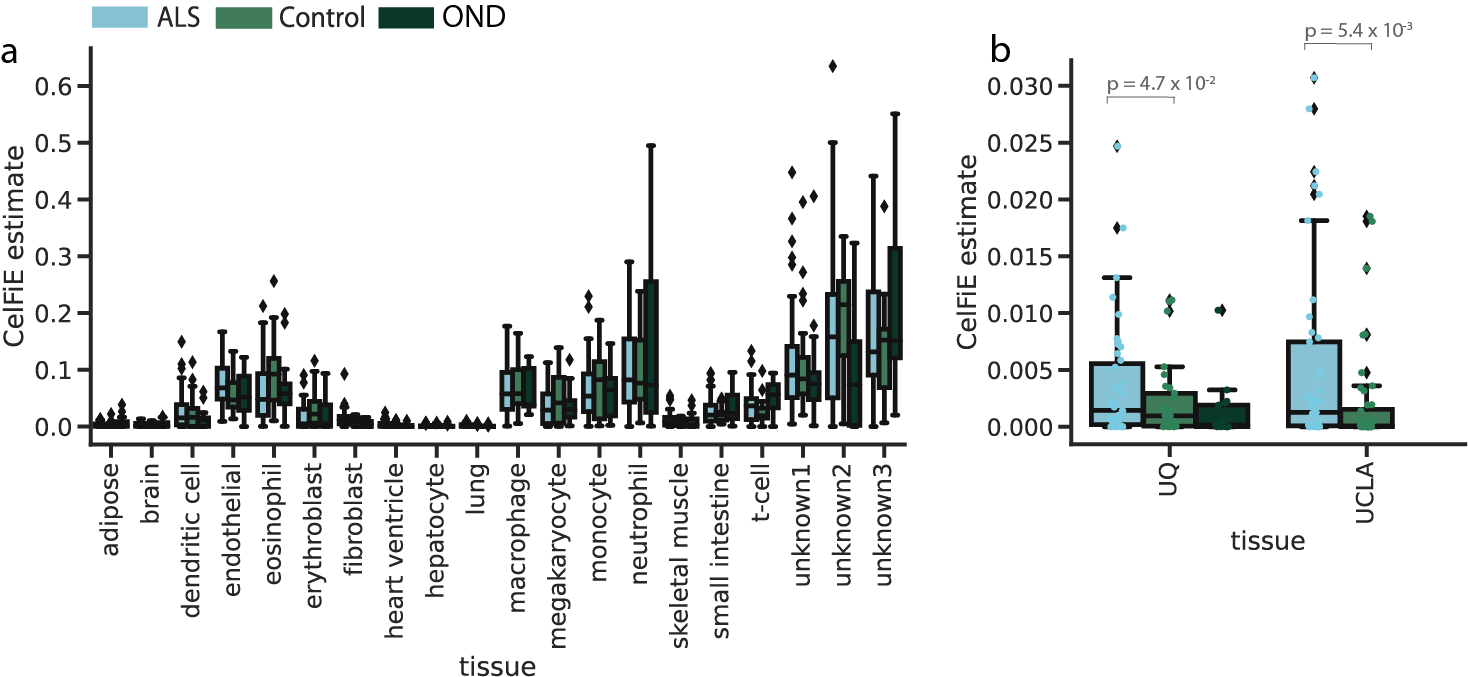
**

#####

##### **Figure S5:** ***Cell-type decomposition estimates.* (a)** The proportion of cfDNA estimated by CelFiE to originate from each tissue for each sample type. (b) The CelFiE estimate of heart ventricle for each sample type in each cohort.


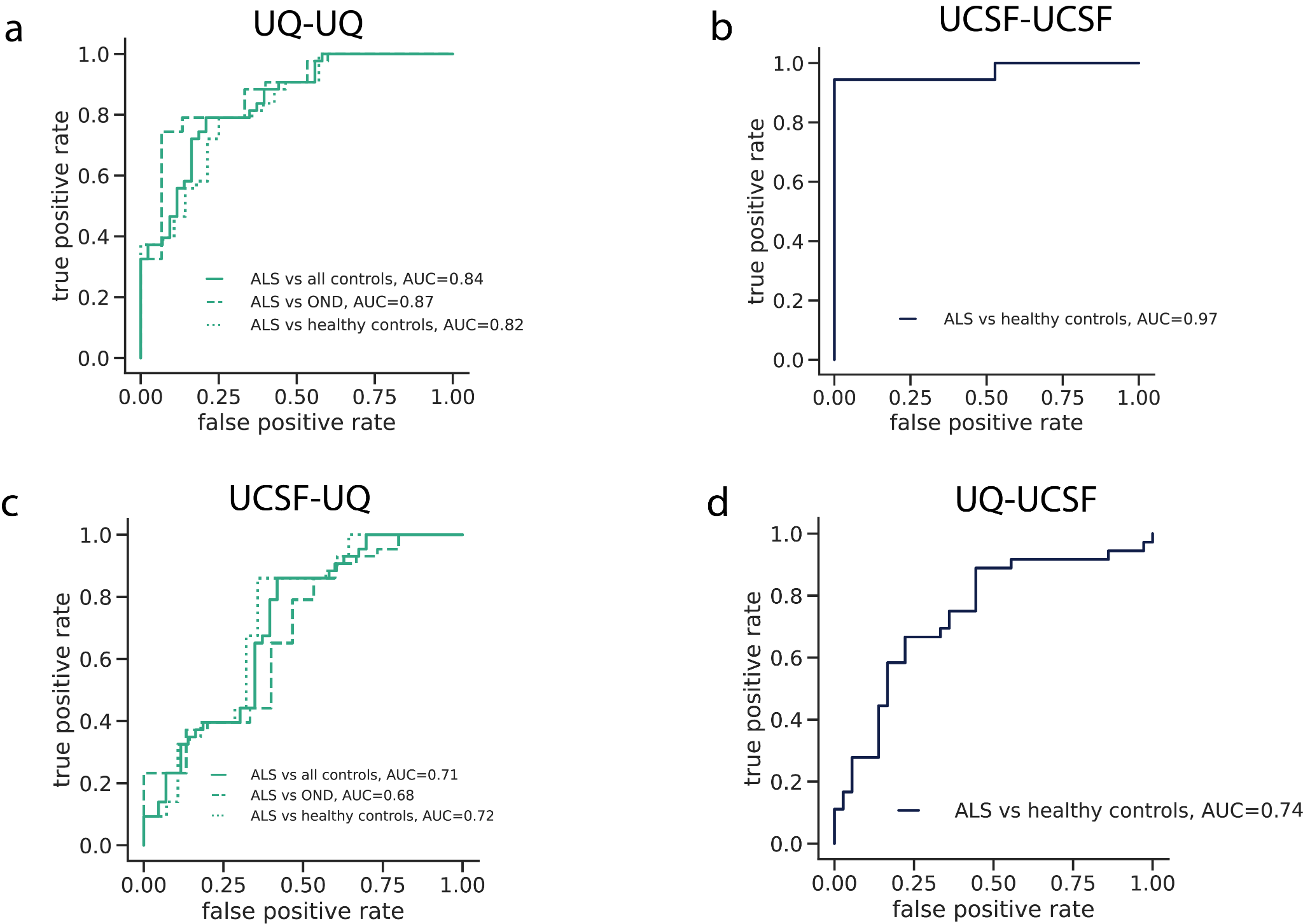


##### **Figure S6: *ALS disease classification using CpG coverage.*** The false positive rate versus true positive rate for models trained and tested using only CpG coverage as input features **and no covariate information** for (a) ten fold cross validation within UQ samples (b) ten fold cross validation within UCSF samples (c) trained on UCSF data and tested on UQ data, and (d) trained on UQ data and tested on UCSF data


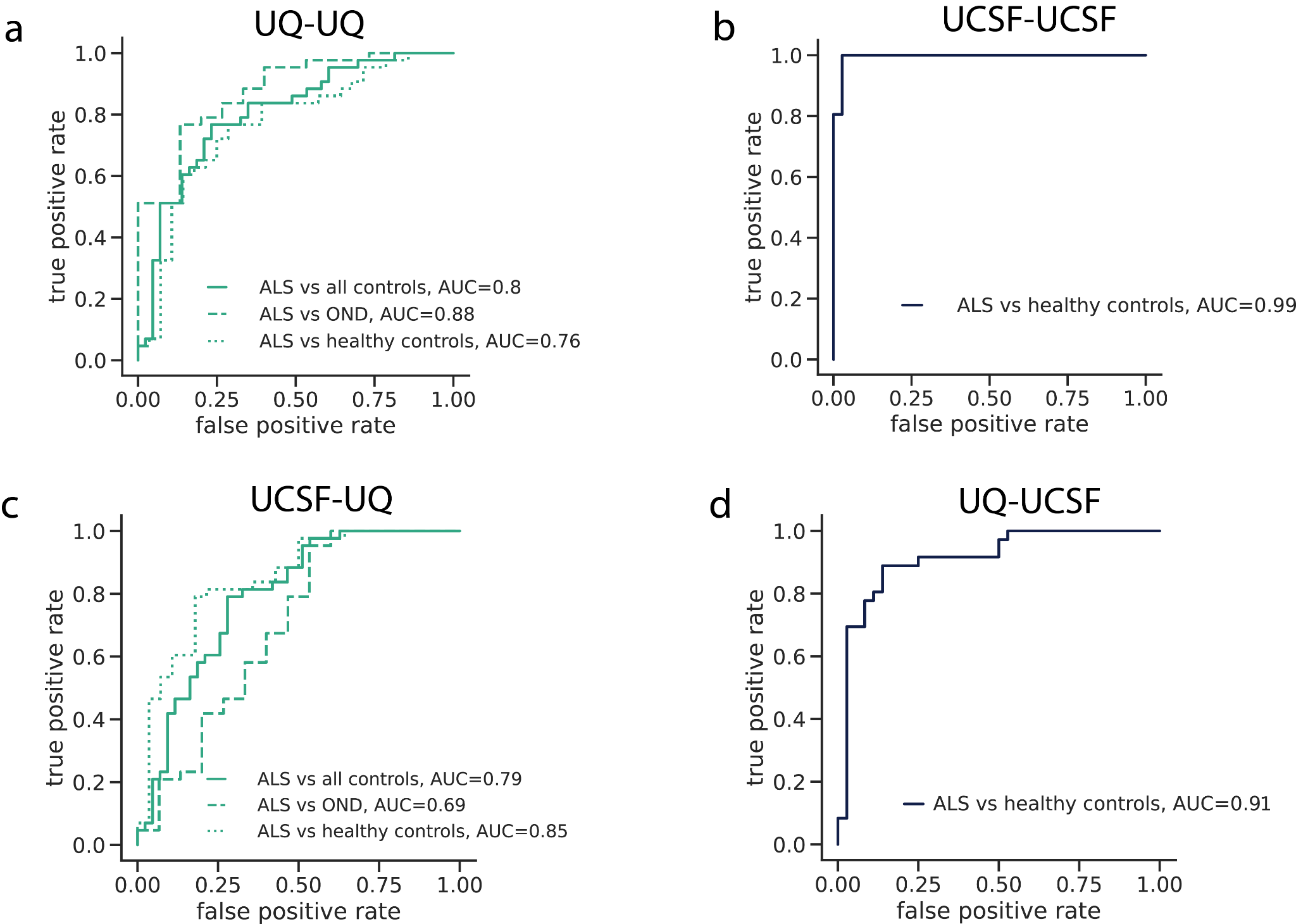


##### **Figure S7: *ALS disease classification using CpG methylation.*** The false positive rate versus true positive rate for models trained and tested using only CpG methylation proportion as input features **and no covariate information** for **(a)** ten fold cross validation within UQ samples **(b)** ten fold cross validation within UCSF samples **(c)** trained on UCSF data and tested on UQ data, and **(d)** trained on UQ data and tested on UCSF data

#####
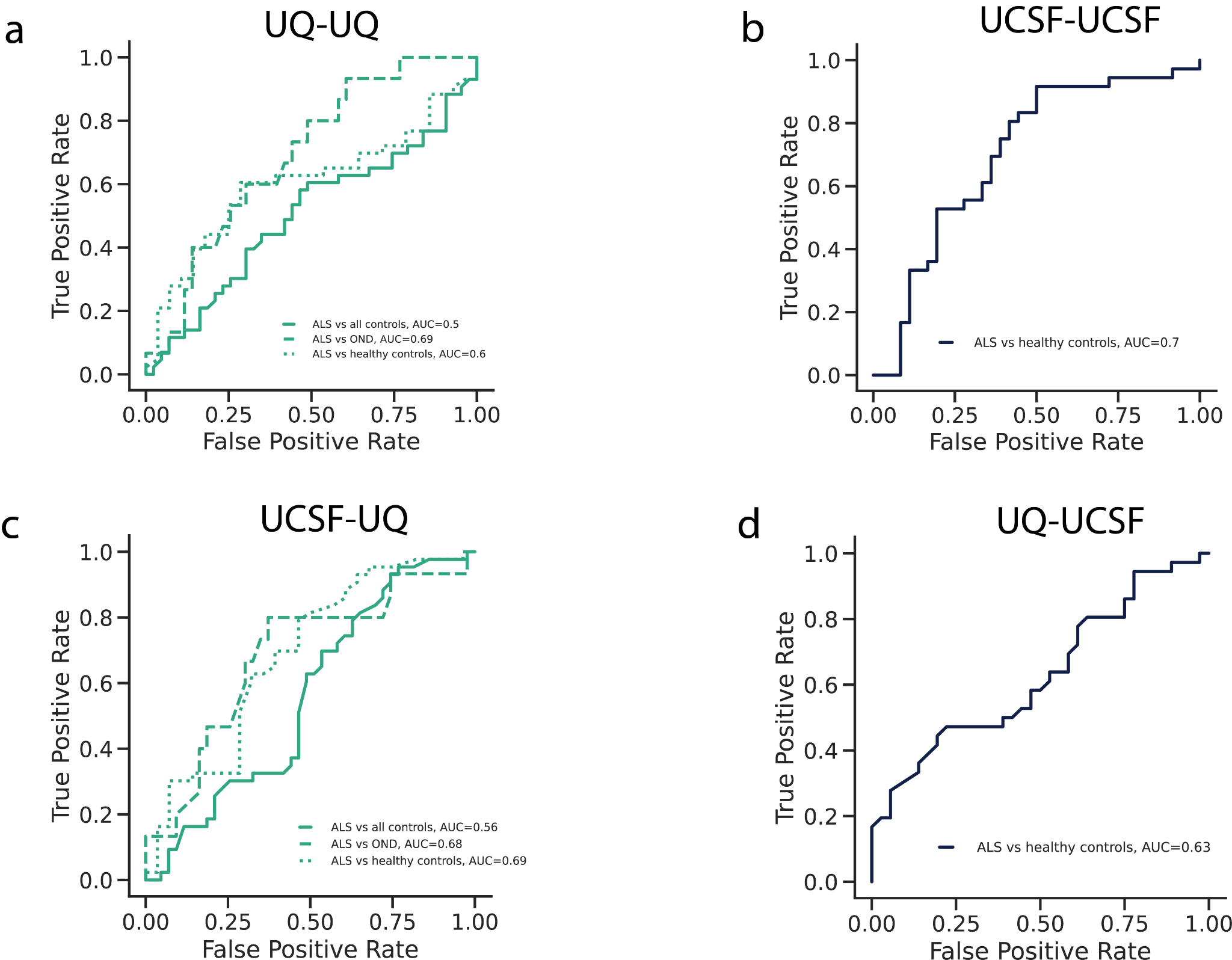


##### **Figure S8: *ALS disease classification using only covariate information.*** The false positive rate versus true positive rate for models trained and tested using only covariate information (age, sex, and SIRE) as input features for **(a)** ten fold cross validation within UQ samples **(b)** ten fold cross validation within UCSF samples **(c)** trained on UCSF data and tested on UQ data, and **(d)** trained on UQ data and tested on UCSF data

##### **
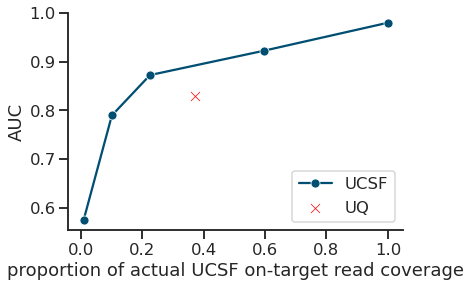
**

##### **Figure S9: *The relationship between read coverage and predictive performance.*** For UCSF cfDNA samples, the total number of reads was randomly downsampled to reduce overall on-target CpG coverage relative to the actual UCSF read coverage. The downsampled samples were then used as input for elastic net models trained using 10 fold cross validation to predict case-control status in the UCSF cohort and the AUC was recorded. The within-cohort UQ AUC is indicated by a red X.

##### **
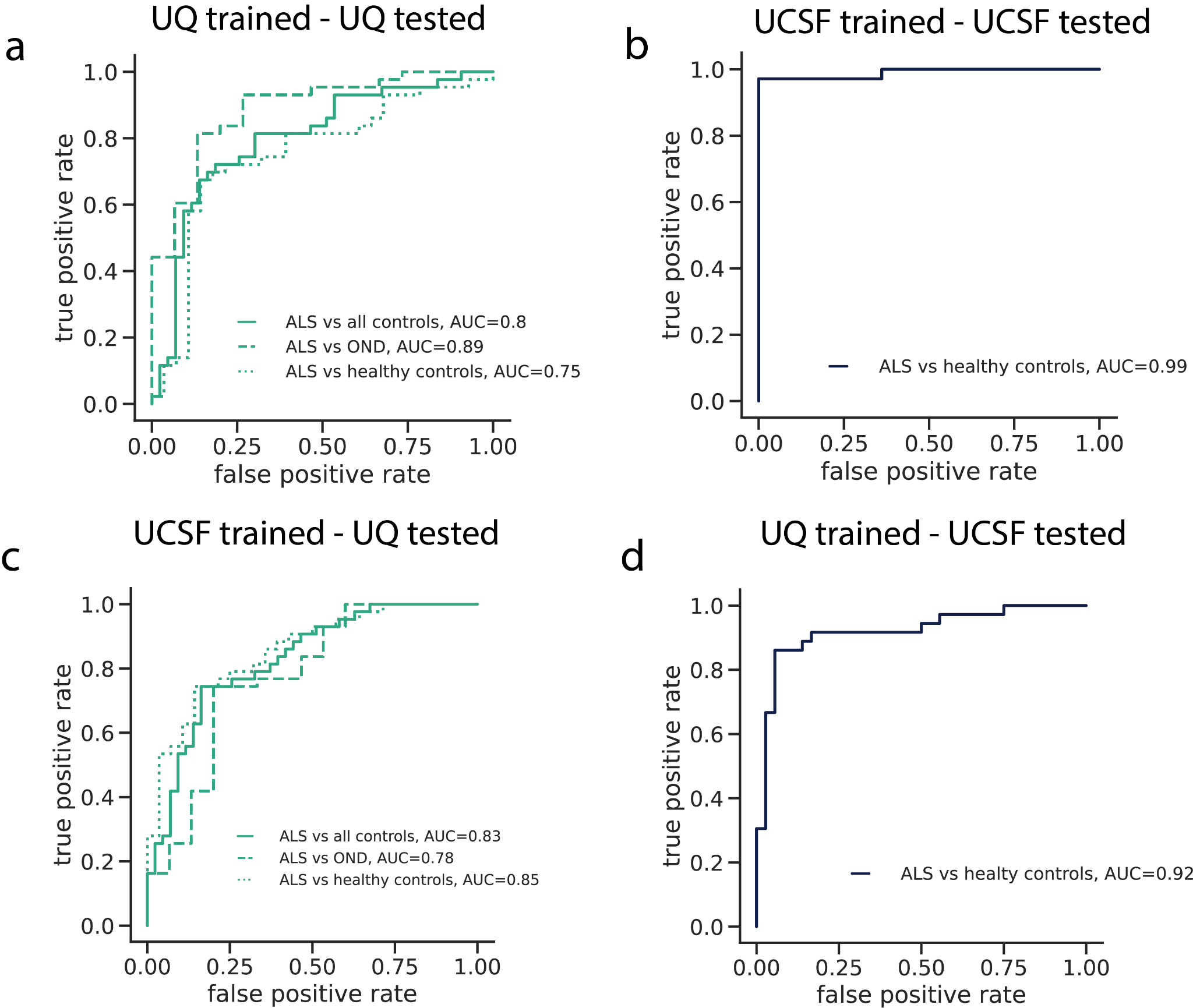
**

##### **Figure S10: *ALS disease classification without skeletal muscle TIMS .*** The false positive rate versus true positive rate for models trained and tested using cfDNA CpG methylation, CpG coverage, and covariate information (age, sex, SIRE, starting cfDNA concentration, and total cfDNA input) for all TIMs besides those chosen for skeletal muscle as input features for **(a)** ten fold cross validation within UQ samples **(b)** ten fold cross validation within UCSF samples **(c)** trained on UCSF data and tested on UQ data, and **(d)** trained on UQ data and tested on UCSF data

#####


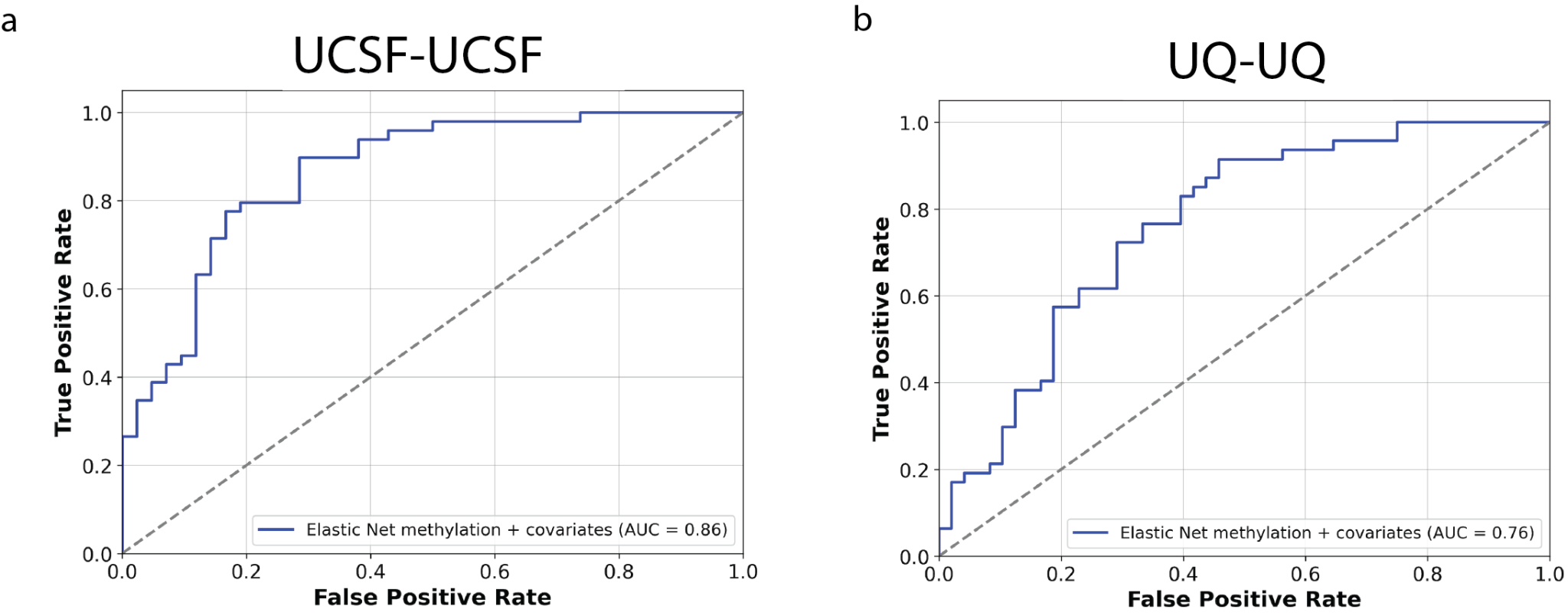


##### **Figure S11: *ALS disease classification with off-target CpGs .*** The false positive rate versus true positive rate for models trained and tested using off target cfDNA CpG methylation trained and tested used **(a)** ten fold cross validation within UCSF samples **(b)** ten fold cross validation within UQ samples.


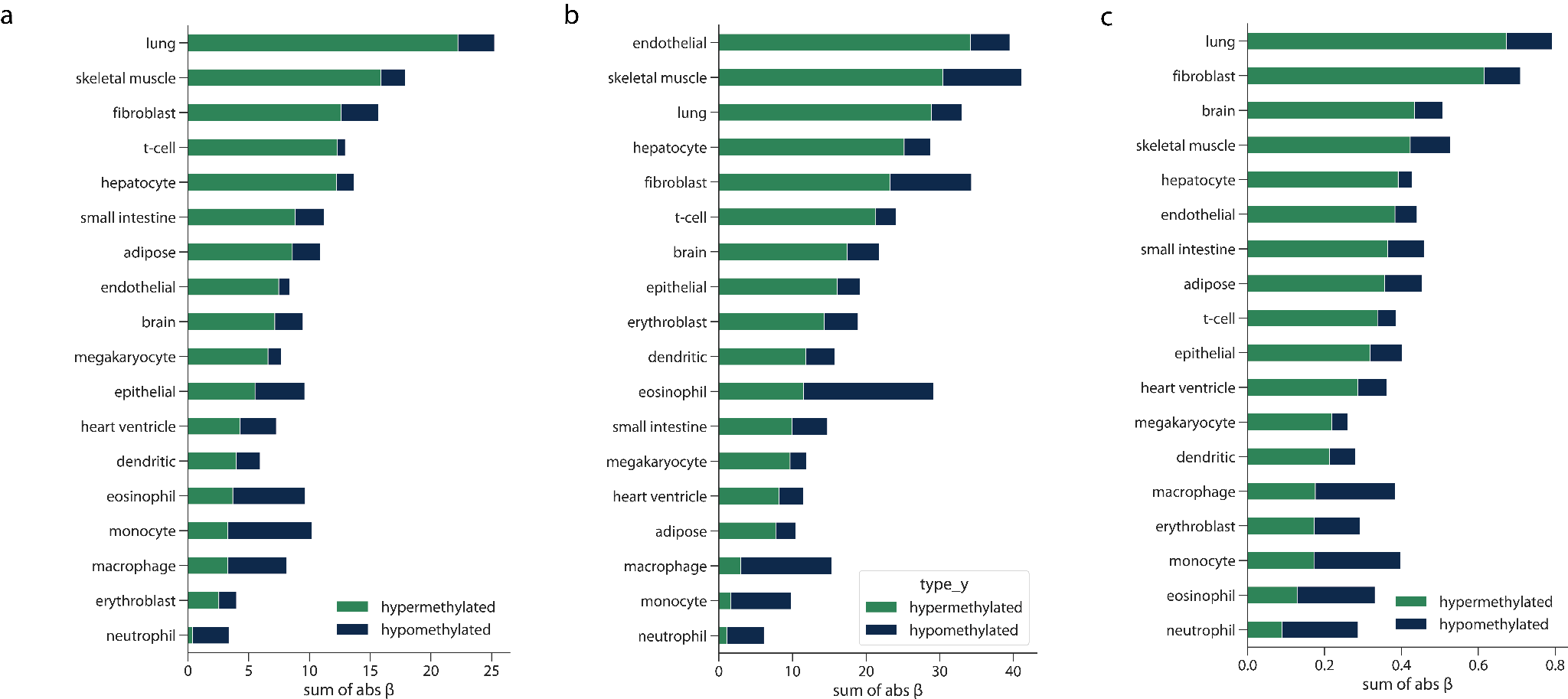


##### **Figure S12: *Weight of features predicting ALS phenotypes.*** For each tissue the TIMs were selected for, and for the type of TIM, the total absolute β value for (**a)** ALSFRS-R (**b)** FVC and (**c)** ALSFRS-R slope phenotypes. A larger absolute β sum indicated that the feature type contributed more to model predictions.


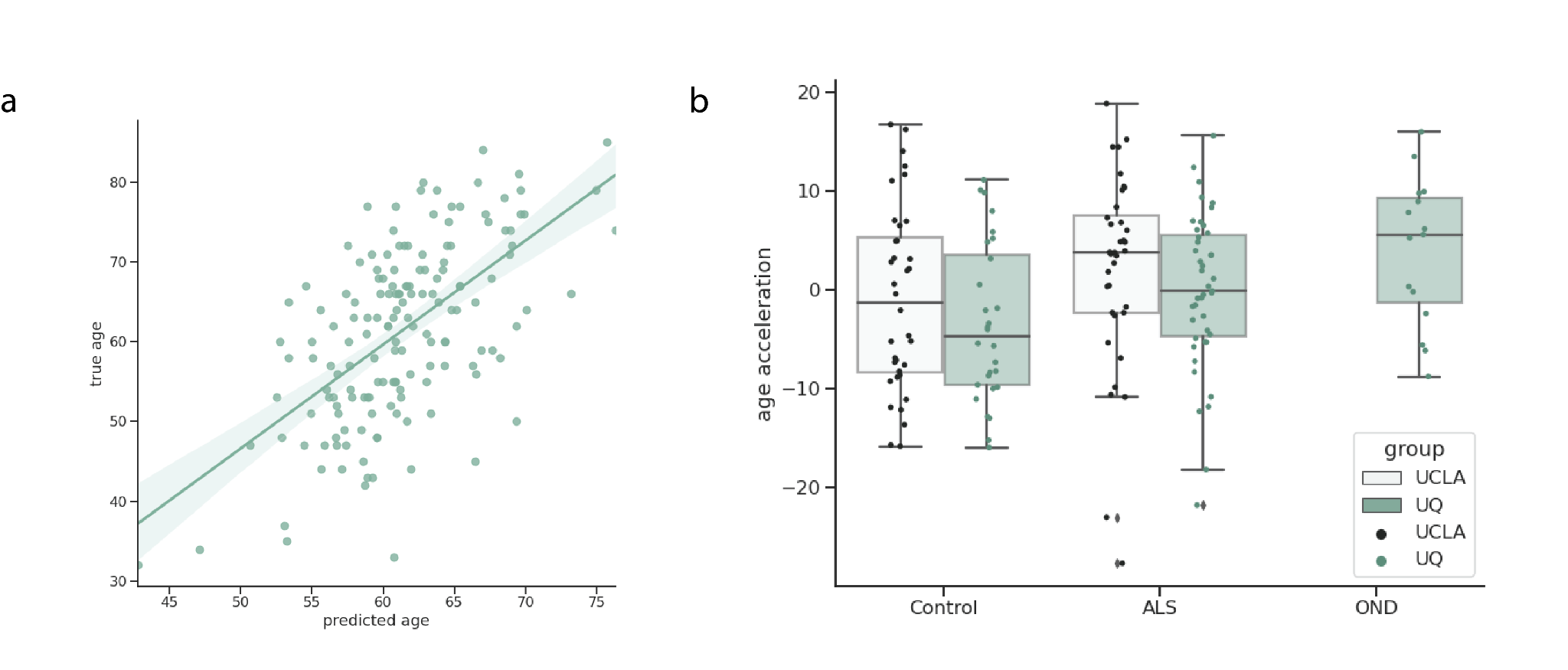


##### **Figure S13: *Epigenetic age acceleration in ALS.*** (**a**) The association between predicted age via DNA methylation and the true age of participants across the UQ and UCSF cohorts. (**b**) The age acceleration of ALS patients, healthy controls, and OND patients.

#### 
